# Patients’ Perspectives on Artificial Intelligence and Digital Transformation in Dental Practice: A Cross-Sectional Study from Romania

**DOI:** 10.3390/dj14090572

**Published:** 2026-09-07

**Authors:** Alin Flavius Cozmescu, Ana Cernega, Andreea Cristiana Didilescu, Marina Meleșcanu Imre, Cristian Funieru, Silviu-Mirel Pițuru

**Affiliations:** 1 Department of Organization, Professional Legislation and Management of the Dental Office, Faculty of Dental Medicine, “Carol Davila” University of Medicine and Pharmacy, 17-23 Plevnei Street, 020021 Bucharest, Romania; alin-flavius.cozmescu@drd.umfcd.ro (A.F.C.); silviu.pituru@umfcd.ro (S.-M.P.); 2 Department of Embryology and Microbiology, Faculty of Dentistry, The “Carol Davila” University of Medicine and Pharmacy, 050474 Bucharest, Romania; andreea.didilescu@umfcd.ro; 3 Department of Prosthodontics, Faculty of Dental Medicine, “Carol Davila” University of Medicine and Pharmacy, 17-23 Calea Plevnei, 010221 Bucharest, Romania; marina.imre@umfcd.ro; 4 Department of Preventive Dentistry, Faculty of Dentistry, “Carol Davila” University of Medicine and Pharmacy, 050037 Bucharest, Romania; cristian.funieru@umfcd.ro

**Keywords:** artificial intelligence, dental patients, digital transformation, doctor-patient relationship, data security, healthcare digitalization, patient perception, informational VUCA

## Abstract

**Background/Objectives:** The integration of artificial intelligence (AI) and digital technologies into dental practice is reshaping clinical workflows, administrative processes, and, increasingly, the patient experience and the doctor–patient relationship. While prior research has documented the attitudes of clinicians and practice managers, the perspective of the patient remains comparatively underexplored. This study examined how dental patients perceive AI integration and digital tools across the dental care pathway, together with the associated implications for data security, cost, and the human dimension of care. **Methods:** A cross-sectional, questionnaire-based study was conducted among 200 dental patients in Bucharest, Romania, and the surrounding region. The instrument assessed perceived difficulty and availability regarding digital technology, current use of digital tools, demographic and educational characteristics (age, gender, practice environment, educational level), and two attitudinal dimensions, namely digital prudence and concern for technological sustainability, across five subdomains of the dental care pathway: scheduling, diagnosis, treatment planning, feedback, and follow-up (dispensarization). Responses were analyzed using non-parametric tests and exploratory principal component analysis with internal-consistency validation. **Results:** Patients expressed moderate-to-high interest in AI support during the diagnostic (median = 3.3, IQR = 2.7–3.9) and feedback (median = 3.11, IQR = 2.78–3.67) stages and the lowest interest in scheduling (median = 2.7, IQR = 2.0–3.3). A marked level of digital prudence was observed (median = 3.24, IQR = 2.82–3.61), reflecting concerns about data security, automation, and a possible weakening of the clinician–patient bond. Younger and academically educated patients reported lower perceived difficulty, higher availability, and greater current use of digital tools (all *p* ≤ 0.001); counterintuitively, the same patients scored significantly higher on digital prudence (Spearman’s ρ = −0.260, *p* < 0.001). Greater familiarity with digital tools was therefore accompanied by a more critical awareness of their informational risks rather than by uncritical acceptance. **Conclusions:** Dental patients approach AI through a dual lens of openness and informed caution, welcoming efficiency gains in the clinical and continuity-of-care stages while voicing measured concerns about data security, affordability, and the preservation of human contact. To interpret this profile, we propose two conceptual contributions: a mapping of patient needs onto Maslow’s hierarchy in the context of AI-mediated care and the Informational VUCA framework, which characterizes the volatility, uncertainty, complexity, and ambiguity that patients face when navigating AI-generated information. The findings point to a clear practical agenda of transparent communication, robust data governance, and education strategies adapted to patients’ educational and demographic profiles, so that AI-enhanced workflows strengthen rather than erode the doctor–patient relationship.

## 1. Introduction

Artificial intelligence (AI) has rapidly evolved from a theoretical construct into a practical instrument that reshapes the architecture of healthcare delivery. Originally conceptualized in 1950 through Alan Turing’s seminal proposal of the test that bears his name, and formally legitimized as an autonomous research field by John McCarthy at the Dartmouth Conference in 1956, AI has progressively migrated from abstract mathematical reasoning into the operational core of medical systems [1,2]. The convergence of computational power, large-scale digitization of clinical data, and refinement of machine learning architectures has enabled AI to support diagnostic accuracy, therapeutic planning, predictive analytics, and patient communication across virtually all medical specialties, including dentistry [3,4,5,6,7]. In contemporary dental practice, AI-driven tools are increasingly integrated into workflows ranging from radiographic interpretation and caries detection to implant planning, prosthetic design, and patient management platforms [8,9,10,11,12]. A growing body of survey research has examined dental professionals’ readiness, attitudes, knowledge, and perceived barriers regarding AI adoption [13,14,15,16], whereas the patient’s perspective has remained comparatively underexplored. The integration of AI into dental medicine, however, cannot be understood as a purely technical transition. It represents a multidimensional transformation that simultaneously affects three interdependent actors of the dental healthcare system: the dentist, who anchors clinical expertise and therapeutic decision-making; the dental practice manager, who orchestrates the administrative and organizational architecture of care delivery; and the patient, who is the ultimate beneficiary of, and active participant in, the medical act [17,18]. Within this triadic structure, the present research is the third installment of a coordinated investigation conducted by our team, complementing two previous studies that examined the perspectives of Romanian dental clinicians [19] and dental clinic managers [20] on the integration of AI and digital workflows in routine practice. Together, these three components are intended to provide a comprehensive picture of how digital transformation is perceived and operationalized across the entire dental healthcare ecosystem, with the patient’s perspective representing the indispensable final piece of the puzzle.

The patient’s role within the doctor–patient relationship has evolved substantially over the past decades. From a predominantly passive recipient of medical services, the patient has progressively assumed the role of an informed partner and decision-maker, in accordance with the principle of autonomy formalized through informed consent procedures and reinforced by contemporary patient rights legislation [21,22]. This evolution has been accelerated by the digital revolution and, more recently, by the widespread accessibility of AI-based information platforms, which allow patients to seek, compare, and even anticipate medical information prior to consulting a clinician [23,24]. Conversational AI systems and health chatbots have become increasingly prominent channels through which patients obtain such information [25,26], although the accuracy and potential bias of these outputs remain difficult for non-specialists to appraise [27]. The result is a more empowered, but also more demanding, patient who arrives at the dental practice with pre-formed expectations and, frequently, with a self-formulated understanding of their condition. While this dynamic may strengthen patient engagement and adherence, it also introduces new tensions within the clinician–patient relationship, particularly when AI-generated information conflicts with the clinician’s evidence-based assessment [28,29].

This transformation does not only reshape the informational balance between clinician and patient; it also requires a renewed theoretical understanding of the doctor–patient relationship itself. In dentistry, where clinical decisions often combine biological, functional, esthetic, financial, and psychological considerations, the interaction between dentist and patient becomes particularly sensitive to changes in knowledge asymmetry, expectations, and perceived authority. Therefore, before examining how AI may reshape patient attitudes and expectations, it is necessary to revisit the conceptual foundations of the doctor–patient relationship and the reciprocal roles that structure it.

**Figure 1:**
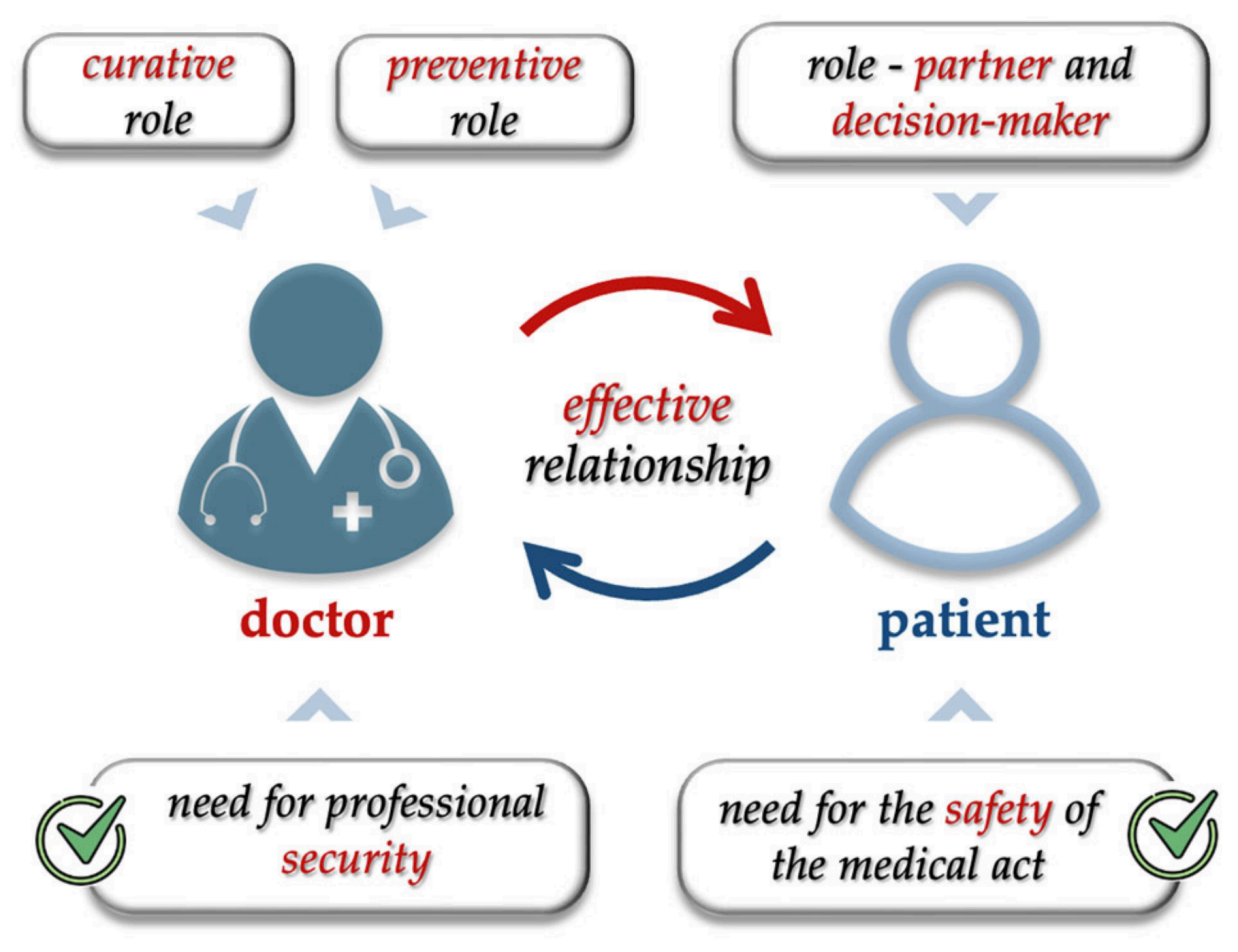
Doctor–patient relationship—key element of the healthcare system.

The specialized literature offers a wide range of interpretations of the doctor–patient relationship and how it should function. However, one clear certainty remains: the doctor and the patient together form a key element of the healthcare system. This relationship represents the fundamental unit that ensures the functionality of the system, a relationship defined by a set of specific needs that must be met in order to generate mutual benefits for both actors (Figure 1).

Before addressing the needs that generate mutual benefits, it is essential to clarify the role and mission of each actor involved. The primary objective lies in the need to preserve the most important social and legal values: human life and health. In this context, the physician’s role must be viewed from both a *curative perspective*—performing the medical procedures necessary for the patient’s recovery—and a *preventive* one, which is essential to maintaining health. This includes educating and informing the patient, a prerogative granted through the physician’s acquired competencies and skills. As for the patient, they are the central subject of the medical act, acting both as a partner and as a decision-maker in the treatment process, in accordance with the principle of autonomy, which is reflected in the right to accept or refuse the proposed treatment. This intersection of roles enables the achievement of the relationship’s core objective, which ultimately derives from a well-defined patient need—*the rational need to improve health status and ensure the safety of the therapeutic act*. As a result of achieving this objective and addressing the patient’s rational need, a corresponding need is fulfilled, generating a benefit for the physician—namely, the minimization of conflict risk and the enhancement of professional and reputational security. In this context, at the intersection where the needs of both actors are met, we can speak of the construction and effective management of the doctor–patient relationship.

Against this background, the integration of AI into dental care should not be understood solely as a technological or operational development, but as a factor capable of influencing the very architecture of the doctor–patient relationship. If the therapeutic relationship is built upon the convergence of patient needs, professional responsibility, trust, and shared decision-making, then any technology that modifies access to information, clinical communication, or treatment expectations must be examined through a framework that accounts for both rational and relational dimensions. This is particularly relevant when assessing patients’ perceptions of AI, since acceptance or resistance may depend not only on perceived usefulness, but also on whether the technology is seen as supporting or disrupting fundamental human needs within the therapeutic encounter.

The research question proposed in this study is therefore “*From the patient’s perspective, how are demographic, educational, and contextual factors associated with the perception of AI and digital workflow integration in dental care, and how do these perceptions relate to patients’ trust, expectations, and the human dimension of the doctor–patient relationship?*”

## 2. Materials and Methods

The Materials and Methods Section outlines the study architecture, participant eligibility and recruitment strategy, measurement instruments, procedural sequence, and analytical approach. To ensure methodological transparency and rigor, reporting adhered to the STROBE checklist for cross-sectional investigations. We implemented a quantitative, cross-sectional survey targeting dental patients, aimed at characterizing their perceptions, attitudes, and availability regarding the integration of digitalization and artificial intelligence into the dental care pathway (e.g., appointment scheduling, diagnosis, treatment planning, feedback collection, and follow-up management). For the purposes of this study, the digital dentistry component was operationalized across the same five workflow-related subdomains used in the previous two studies of our research series [19,20], namely scheduling, diagnosis, treatment planning, feedback, and follow-up (dispensarization—the term used in several healthcare systems for the structured, long-term monitoring and periodic recall of patients)—to ensure methodological consistency and comparability across the three perspectives of the dental healthcare triad. Given this cross-sectional design, the study is intended to characterize the patient perceptions and attitudes actually measured through the questionnaire and to identify associations among these variables, rather than to establish causal or mechanistic relationships between the investigated factors and patient outcomes. In this study, “perceived difficulty” denotes the respondent’s perceived effort/complexity associated with using digital technology in interactions with dental services, while “availability” refers to the respondent’s perceived willingness to adopt new AI-based and digital solutions within their dental care pathway. This patient-centric framing captures the experiential and relational dynamics of technology adoption, complementing the previously published clinician [19] and managerial [20] perspectives and enabling inference about how digital tools and AI reshape the patient experience and the doctor–patient relationship.

Statistical analysis was performed using IBM SPSS Statistics for Windows, version 25 (IBM Corp., Armonk, NY, USA) and Microsoft Office Excel/Word 2024. Quantitative variables were reported as means with standard deviations or medians with interpercentile ranges; their distribution was assessed using the Shapiro–Wilk test. Non-parametric independent quantitative variables were compared between investigated factors via Mann–Whitney U/Kruskal–Wallis H tests (with Dunn–Bonferroni post-hoc tests), while correlations between non-parametric independent quantitative variables were evaluated using Spearman’s rho correlation coefficient. Qualitative variables were expressed as absolute values or percentages, and intergroup differences were tested with Fisher’s Exact Test. Z-tests with Bonferroni corrections provided further detail in contingency tables.

The investigated subdomains were as follows: scheduling, diagnosis, treatment planning, feedback, and follow-up (dispensarization), each containing 15 items in the survey. In each of the subdomains, exploratory Principal Component Analysis (PCA) models were used, retaining the first component with the highest explained variance (largest eigenvalue, λ) and with items included in the first component having at least an absolute loading of ≥0.30. Subsequently, extracted items were reintroduced into the PCA for validation (based on Bartlett’s Test of Sphericity). Analyzing all items in the survey, an overall exploratory PCA was conducted to observe the overall digitalization scores, in which two components emerged.

The patient questionnaire was developed as one of three parallel instruments within a single coordinated research project on AI and digital transformation in dental care, alongside the instruments addressed to dental clinicians [19] and dental clinic managers [20]. The three questionnaires were designed together and administered over the same period, with data collection carried out in parallel across the three respondent groups. All three instruments were built around the same five workflow-related subdomains of the dental care pathway, with the patient version worded to reflect the patient’s perspective rather than that of clinicians or managers. Item content was grounded in the established stages of the patient’s clinical journey and in the shared thematic framework of the project. Content coverage was therefore supported by this common, deliberately comparable design and by the instrument’s alignment with the recognized structure of the dental care pathway. Consistent with the exploratory, pilot character of the study, no formal expert-panel review, independent pilot testing, or large-scale cross-cultural psychometric validation was performed; the psychometric assessment was limited to the internal-consistency and factor-structure analyses described below.

Each item received a score from 1 (minimum interest) to 5 (maximum interest). Because the components extracted from the overall PCA comprised items drawn from all five subdomains and from all three item sets (challenges, ways of improvement, and difficulties in usage), the constituent items of each component were examined individually and collectively at a conceptual level before any composite score was computed. This step was necessary in order to establish the substantive meaning of each component and, consequently, the direction in which each item contributed to it. Items whose wording expressed the opposite conceptual position to that of the emerging construct were reverse-scored, so that all items contributing to a given score would be oriented in the same conceptual direction before averaging. We wish to state explicitly that the sign of the component loading was not treated as the justification for this decision, since the direction of principal-component loadings is mathematically arbitrary; the loading sign served only as the initial signal that prompted the examination of the item, and in every case the decision to reverse an item was taken on the basis of its content. In the present data the two criteria converged, in that each negatively loading item was also, on inspection of its wording, conceptually opposite in direction to the positively loading items of the same component. Internal consistency, reported below as Cronbach’s alpha, was computed after this reorientation and was examined together with the alpha-if-item-deleted values for every constituent item. The overall score was the arithmetic mean of all included items. PCA was used in a strictly exploratory capacity, to examine item-loading patterns and to identify the items requiring conceptual reorientation; we emphasize that PCA was used only for this purpose and not as evidence of construct validity. The adequacy of the correlation matrix for this exploratory analysis was assessed with Bartlett’s Test of Sphericity, which does not by itself validate the construct or the scoring structure. The internal consistency of the resulting scores was assessed via Cronbach’s alpha, examining changes in the coefficient upon item removal. These procedures constitute a preliminary, exploratory assessment of internal consistency and dimensionality rather than a formal psychometric validation of the instrument; this is acknowledged among the study’s limitations. Statistical significance was set at α = 0.05.

Given the exploratory, pilot nature of the study and the limited size of several outcome subgroups, the analytical plan was deliberately restricted to bivariate comparisons; adjusted multivariable models were not fitted, as the available cell counts would not support stable estimation (see Limitations).

To prevent conceptual overlap between the various terms used throughout this study, we distinguish three levels of analysis. First, the directly measured operational variables, obtained from patient responses, comprise perceived difficulty, availability (the patient’s perceived willingness to adopt new AI-based and digital solutions; the terms “availability” and “willingness to adopt/use” are employed as equivalents in this study), current use of digital tools, and the interest scores computed for each of the five care-pathway subdomains. Second, two composite attitudinal scores—the digital prudence score and the technological sustainability score—were derived empirically through exploratory principal component analysis and assessed for internal consistency via Cronbach’s alpha; these are measured constructs that summarize patterns across multiple items rather than single responses. Third, the Maslow-based hierarchy of patient needs and the proposed Informational VUCA framework are interpretative constructs, introduced in the Discussion to organize and explain the empirical findings; they are conceptual lenses rather than directly measured quantities. Throughout the manuscript, the digital prudence and technological sustainability scores are reported as measured variables in the Results and subsequently used as interpretative anchors in the Discussion, and we have aimed to keep this dual role explicit wherever these terms appear.

The main eligibility criteria were dental patients aged 18 years or older, currently receiving or having recently received dental care in dental practices located in Bucharest and surrounding areas, who were willing to participate and complete the questionnaire. There were no restrictions on gender, educational level, or socio-economic status. Individuals who had never received dental care or who were unable to provide informed consent were excluded.

Participants were recruited using a purposive/convenience sampling approach between October 2024 and March 2025. The questionnaire was administered in paper-based format and was distributed in person by the research team to eligible adult patients—specifically, active dental patients with recent or ongoing dental care and regular attendance at dental practices in Bucharest and the surrounding areas, including nearby rural communities. Participation was voluntary, and informed consent was obtained prior to participation. Because this purposive, in-person distribution strategy did not involve a closed sampling frame with a defined denominator, the total number of patients approached could not be established, and a formal response rate could not be calculated; this is acknowledged as a limitation of the study.

The study population comprised 200 dental patients across different demographic and educational profiles. Independent variables included demographic and educational attributes: age and level of education (operationalized as academic education (bachelor’s degree or higher) versus non-academic education (below bachelor’s degree), given the observed concentration of responses around the higher-education threshold). This framework sought to provide a nuanced understanding of the factors influencing patient acceptance of AI and digital technologies in dental care.

Informed consent was obtained prior to data collection, and responses were aggregated and analyzed according to ethical and academic standards. The questionnaire was reviewed and approved by the Scientific Research Ethics Committee of the Carol Davila University of Medicine and Pharmacy in Bucharest (code PO-35-F-03, no. 28.287 of 1 October 2024).

The study was conducted between October 2024 and March 2025. There were no missing data, as all questionnaires were completed in full. No formal sample size calculation was performed, as the research was designed as a pilot, exploratory study focused on patient perceptions of AI integration in the dental care pathway and the implications for the doctor–patient relationship. Limitations include the uneven distribution of respondents by area of residence (with rural patients representing a smaller subgroup), the geographic concentration in Bucharest and adjacent areas, and the rapidly evolving nature of the digital and AI landscape in healthcare. These issues can be addressed in future studies; the present research serves as a starting point and guide for subsequent larger-scale investigations.

## 3. Results and Discussions

### 3.1. Demographic Results

A total of 200 dental patients completed the questionnaire, providing an overview of patient perspectives on digitalization and AI integration across the dental care pathway, including perceived effects on the doctor–patient relationship, expectations regarding diagnostic and therapeutic processes, and concerns related to data security, financial sustainability, and the human dimension of care. Participants were recruited from dental practices located in Bucharest and surrounding areas, including nearby rural communities, capturing a broad range of demographic, educational, and contextual profiles.

The sample showed a balanced gender distribution, with 53.5% men and 46.5% women, and was predominantly urban-based, with 169 participants (84.5%) residing in urban settings and 31 participants (15.5%) in rural settings. The mean age was 40.95 ± 13.94 years, with a median of 39 years (IQR = 30–50), reflecting a broadly distributed adult population. Age distribution showed the following pattern: 18–24 years (10.5%), 25–30 years (16%), 31–35 years (15%), 36–40 years (13.5%), 41–45 years (11%), 46–50 years (9.5%), 51–55 years (7.5%), 56–60 years (6.5%), and >60 years (10.5%). The relatively even spread across age strata, with a notable representation of patients above 60 years (10.5%), allowed for meaningful comparisons between generational groups and represents a methodological strength compared to the more clustered age distributions reported in our previous clinician [19] and managerial [20] studies.

Educational background was diverse and is summarized as follows: primary education (2.5%), secondary education (3.5%), high school (18%), post-high-school studies (7%), bachelor’s degree (39.5%), master’s degree (23%), and doctoral degree (6.5%). For the purposes of analytical comparison, education was operationalized as a binary variable distinguishing academic education (bachelor’s degree or higher, n = 138, 69%) from non-academic education (below bachelor’s degree, n = 62, 31%, comprising primary, secondary, high-school, and post-high-school education). This dichotomization was adopted for three reasons. First, the distribution of responses clustered naturally around the higher-education threshold, making this a meaningful empirical cut point. Second, several of the original educational categories contained too few respondents to support robust subgroup comparisons, particularly primary (2.5%) and secondary (3.5%) education, which would have yielded unstable estimates in contingency-table analyses. Third, the academic versus non-academic distinction is a widely used and readily interpretable operationalization in the health-literacy and digital-health literature. This grouping therefore allowed for a more interpretable and statistically stable analysis of how educational background relates to patient attitudes toward digital and AI-supported dental care.

Overall, the demographic structure of the sample indicates a broadly diverse adult cohort with substantial heterogeneity in age, educational background, and contextual exposure to digital technologies, which is methodologically relevant for interpreting attitudes toward AI-driven workflow transformation in dental practice.

### 3.2. Subdomains of Dental Treatment

Stages such as scheduling, diagnosis, treatment planning, feedback collection, and follow-up (dispensarization) represent the natural touchpoints between the patient and the dental care system, and each of these stages is increasingly being reshaped by digital tools and AI-supported applications. From the patient’s perspective, these subdomains are not only technical or operational stages, but also moments in which trust, communication quality, perceived competence, and the human dimension of care are continuously evaluated and reaffirmed. Maintaining the doctor–patient relationship, ensuring transparency and clarity of information, safeguarding data confidentiality, and preserving the affordability of dental services emerged as central concerns in patients’ responses throughout the questionnaire.

**Figure 2:**
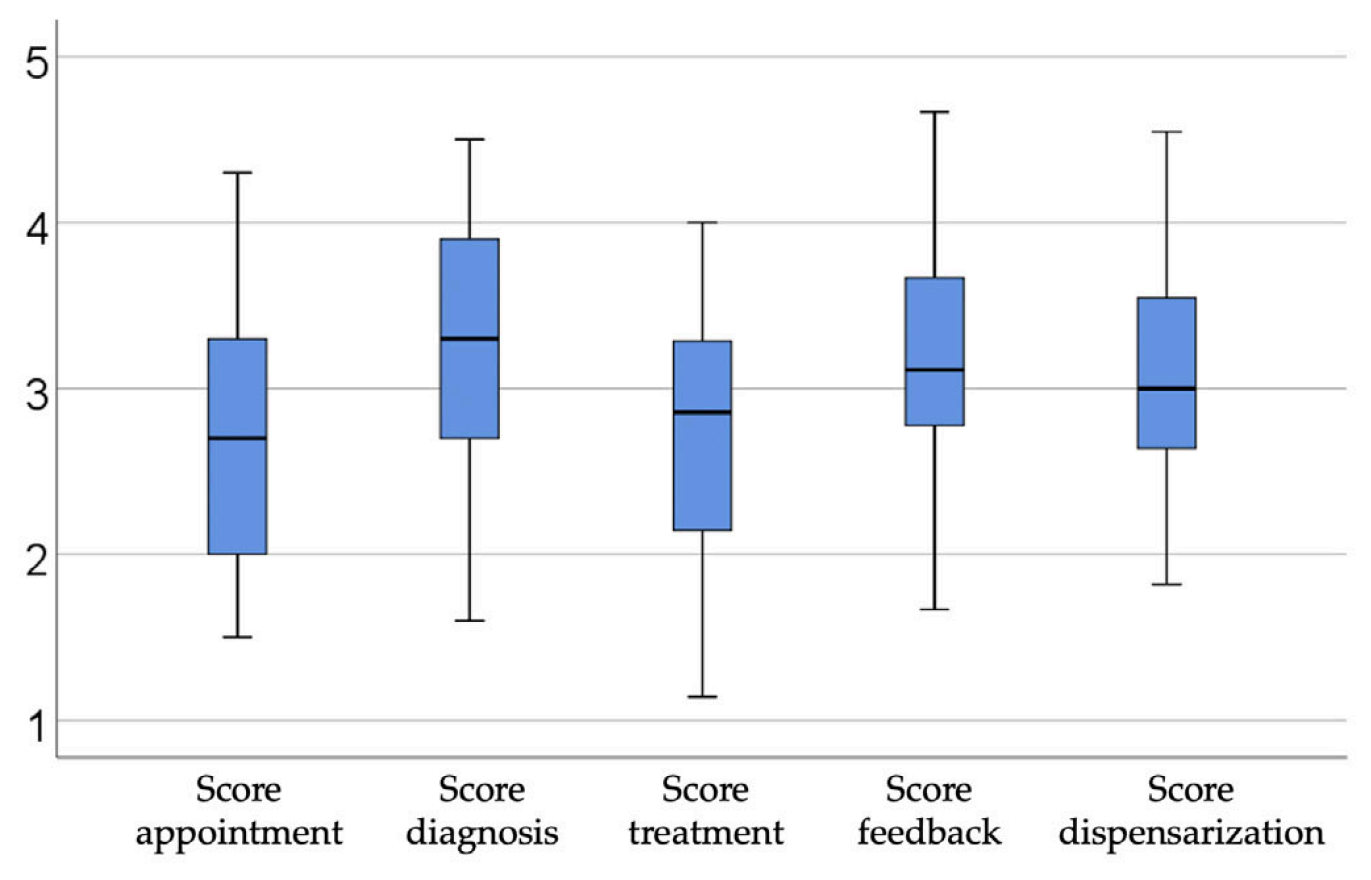
Distribution of interest scores by subdomain among dental patients.

Figure 2 shows the distribution of interest scores by subdomain. The results show that the highest interest scores were observed for diagnosis (median = 3.3, IQR = 2.7–3.9) and feedback (median = 3.11, IQR = 2.78–3.67), where the level of interest was medium to high, while the lowest interest score was for scheduling (median = 2.7, IQR = 2.0–3.3), where the level of interest was medium to low. Treatment planning (median = 2.86, IQR = 2.14–3.29) and follow-up/dispensarization (median = 3.0, IQR = 2.64–3.55) showed intermediate scores, with treatment planning displaying the highest dispersion across responses.

#### 3.2.1. Analysis of the Diagnosis Subdomain

The diagnostic subdomain received the highest level of patient interest in the present study (median = 3.30, IQR = 2.70–3.90). This pattern suggests that patients perceive AI primarily as a credibility-enhancing instrument in the most consequential phase of the dental encounter. From the patient’s perspective, diagnosis is not merely a technical assessment but rather a critical moment in which the clinician’s competence is implicitly evaluated and the foundation of trust for the entire therapeutic process is established [19]. Patients appear to view AI-supported diagnostic tools as resources that may reinforce, rather than replace, the clinician’s professional judgment, functioning as a form of internal validation that strengthens confidence in the proposed care plan. This interpretation is consistent with the broader literature describing AI as a “second-opinion” tool that complements clinical expertise [30,31,32,33] and addresses the patient’s fundamental rational need for diagnostic accuracy and therapeutic safety, a need increasingly substantiated by large-scale, real-world validations of AI diagnostic performance in other areas of medicine [34]. The implication for clinical practice is that diagnostic AI tools should be introduced in ways that are visible to the patient, with transparent communication regarding their role as decision-support resources, in order to preserve the patient’s perception of the clinician’s central authority while leveraging the credibility-enhancing potential of digital tools.

#### 3.2.2. Analysis of the Feedback Subdomain

The feedback subdomain received one of the highest levels of patient interest (median = 3.11, IQR = 2.78–3.67), representing one of the most distinctive findings of the present study. Unlike clinicians [19] and managers [20], who consistently ranked feedback as a lower-priority area, patients clearly perceive the post-treatment phase as a critical moment in their care experience. From a managerial or clinical perspective, feedback may appear as an operational formality with uncertain return on investment. From the patient’s perspective, however, feedback represents an active expression of being heard, recognized, and considered an important participant in the therapeutic process. It is the moment at which the patient’s experience is formally acknowledged and at which the relational symmetry of the doctor–patient encounter is temporarily restored: the patient, who was the recipient of care, now becomes the source of information that shapes future care. AI-supported feedback systems may enhance this dimension by enabling structured, personalized, and easily accessible feedback channels, but they must be designed with explicit attention to the human and relational character of post-treatment communication. Automated feedback tools that feel impersonal or transactional may inadvertently undermine the very recognition need that feedback collection is meant to fulfill. This finding signals a clear practical implication: feedback digitalization should be approached as a relational, not merely operational, intervention.

#### 3.2.3. Analysis of the Follow-Up Subdomain (Dispensarization)

The follow-up (dispensarization) subdomain received a moderate level of patient interest (median = 3.00, IQR = 2.64–3.55). This level of interest indicates recognition of the value of digital tools in supporting continuity of care, preventive monitoring, and timely recall. This stage is structurally well-suited to AI integration because it allows for personalized, asynchronous communication that does not depend on real-time clinician–patient interaction. From the patient’s perspective, follow-up represents the temporal extension of the doctor–patient relationship beyond the immediate clinical encounter, and it addresses several layered needs: safety (long-term oral health monitoring), order (structured care schedules and reminders), and affiliation (continued connection with the dental practice and the clinician). AI-supported follow-up platforms can effectively address all three needs through automated risk stratification, adaptive recall scheduling, and personalized educational content tailored to each patient’s clinical history and behavioral profile. The moderate level of interest observed in our study, rather than indicating reluctance, likely reflects the patient’s expectation that follow-up should remain personal and meaningful, a digital extension of human care, not a substitute for it.

#### 3.2.4. Analysis of the Treatment Planning Subdomain

Treatment planning received a moderate level of patient interest (median = 2.86, IQR = 2.14–3.29) and showed the highest dispersion across responses of any subdomain. This dispersion indicates substantial heterogeneity in patient attitudes toward AI integration in this subdomain. This dispersion is interpretively rich and reflects a genuine tension within the patient’s experience of care. On one side, treatment planning is a stage at which the patient’s need for information and autonomy is most directly engaged: patients want to understand their therapeutic options, the rationale behind the proposed plan, and the alternatives that may be available. AI-supported decision-support tools that can clarify therapeutic alternatives and improve transparency may therefore be welcomed by some patients as instruments of informed decision-making, particularly as AI- and digital-planning applications mature across dental subspecialties such as orthodontics and prosthetic and occlusal planning [35,36,37]. On the other side, treatment planning is also the stage at which the clinician’s expertise and authority are most visibly exercised and at which the patient’s trust in the clinician is most decisively committed. Patients who place strong emphasis on the clinician’s role may view AI-supported planning tools with reservation, fearing that algorithmic recommendations may dilute the personalized, judgment-based character of the therapeutic plan. The practical implication is that AI integration in treatment planning should be approached through hybrid models that allow the patient to engage with digital tools as an optional layer of information, while preserving the clinician’s central role in the formulation and explanation of the therapeutic plan. Importantly, treatment planning is not confined to technical or prosthetic decisions, but also requires careful consideration of the patient’s systemic conditions, ongoing medications, and prescription safety. In this regard, AI-based decision-support systems may assist clinicians in reviewing medication history, flagging potential drug–drug interactions, and supporting safer prescription and perioperative planning, especially in medically complex or polymedicated patients [38,39]. Such applications should, however, be understood strictly as clinician-supervised decision-support functions rather than autonomous recommendation systems, with the clinician retaining full responsibility for validating any AI-generated output and integrating it into the individualized therapeutic plan.

#### 3.2.5. Analysis of the Scheduling Subdomain

Scheduling received the lowest level of patient interest of all five subdomains (median = 2.70, IQR = 2.00–3.30). This finding diverges from the priorities expressed by managers [20], who emphasized scheduling as a cornerstone of operational efficiency. From the patient’s perspective, scheduling appears to be perceived as a logistical and administrative function that, while necessary, does not carry the same relational or clinical significance as the other subdomains. This relative deprioritization may also reflect a preference for retaining human mediation at the gateway of the care pathway, particularly for sensitive cases (urgent appointments, complex coordination, vulnerable patient categories). It is important to emphasize that this lower interest score does not signal indifference, but rather a clear expectation that scheduling automation should be unobtrusive, reliable, and capable of accommodating the human element when needed. Although automated reminders, digital booking, and teledentistry have been shown to reduce missed appointments and improve access to oral care [40,41,42], their patient-side acceptability appears to hinge on preserving this relational safeguard. Hybrid scheduling models that combine automated booking for routine cases with human availability for sensitive situations may therefore represent the most effective implementation approach from the patient’s perspective.

### 3.3. The Perceived Degree of Difficulty

The findings from our study highlight a generally positive perception among dental patients regarding the level of difficulty experienced when utilizing digital tools in their interactions with dental services. Specifically, 119 participants (59.5%) reported a low degree of difficulty, 57 (28.5%) reported a moderate degree of difficulty, and 24 (12%) perceived a high degree of difficulty. This distribution suggests that a majority of patients perceive digital technology as broadly manageable, although a meaningful minority continues to face substantial challenges. A detailed analysis according to age and educational background yielded the following results.

#### 3.3.1. Analysis of the Perception of the Degree of Difficulty in Relation to Age

**Figure 3:**
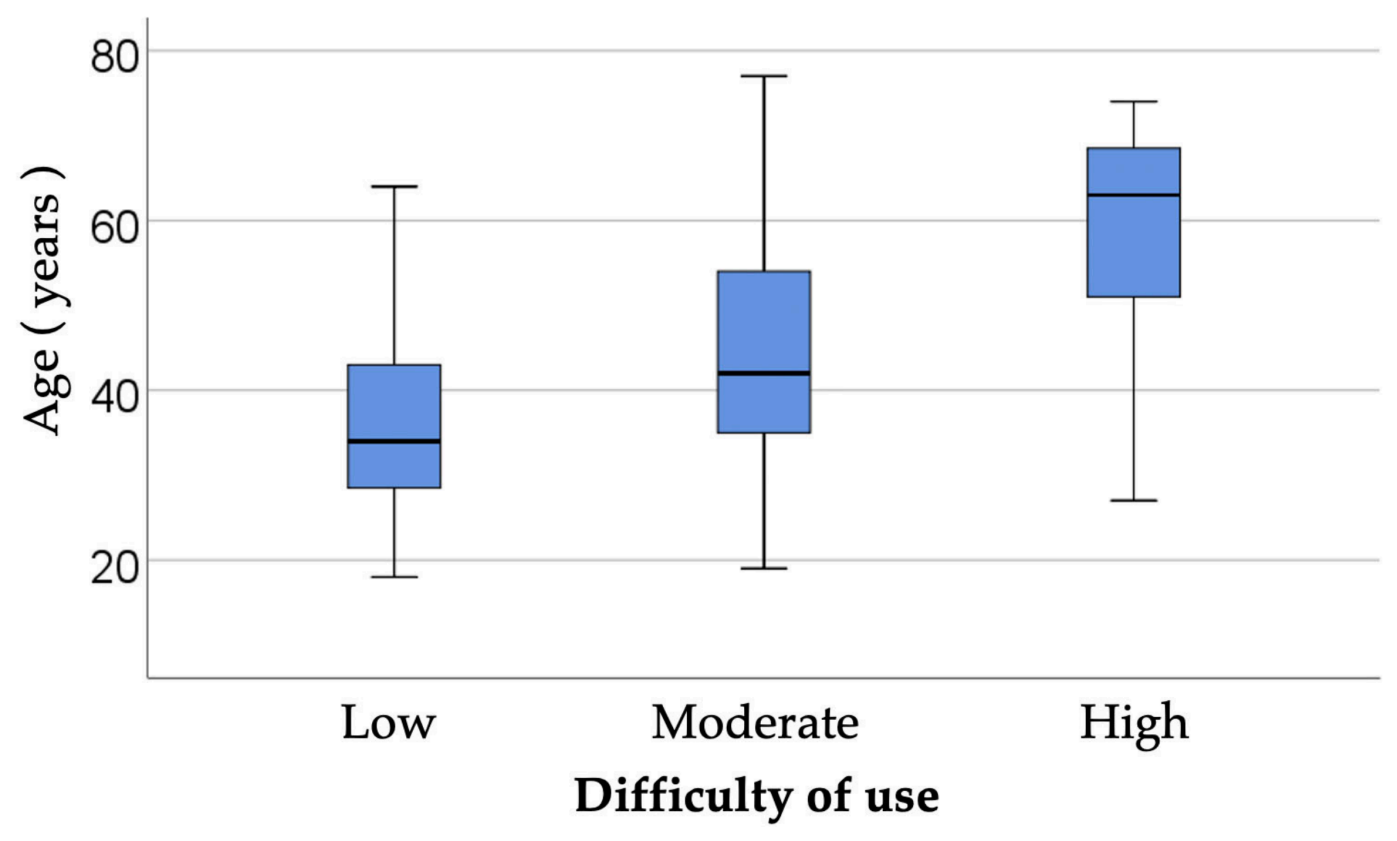
Comparison of patients’ age in relation to perceived degree of difficulty.

The data in Table 1 and Figure 3 represent the comparison of patients’ age in relation to their reported degree of difficulty. The differences observed between groups according to the Kruskal–Wallis H test were significant (*p* < 0.001), and Dunn–Bonferroni post-hoc tests show that patients who reported high difficulty were significantly older (median = 63, IQR = 50.5–68.75) compared to patients who reported moderate difficulty (median = 42, IQR = 35–54.5) (*p* = 0.004) or low difficulty (median = 34, IQR = 28–43) (*p* < 0.001); patients with moderate difficulty were also significantly older than those with low difficulty (*p* < 0.001), (Kruskal–Wallis H(2) = 45.431, *p* < 0.001, ε^2^ = 0.228, a large effect).

Further detail emerged from the categorical age-group analysis (Table 2). According to Fisher’s Exact Test, the differences across age categories were significant (*p* < 0.001). Bonferroni-corrected comparisons of column proportions indicated that patients aged 25–30 years were over-represented among those reporting low difficulty (24.4% of the low-difficulty subgroup vs. 3.5% of the moderate-difficulty subgroup), whereas patients aged over 60 years were over-represented among those reporting high difficulty (54.2% of the high-difficulty subgroup vs. 1.7% and 10.5% of the low- and moderate-difficulty subgroups). Within the over-60 group itself, high difficulty was the predominant response (61.9%).

These results indicate that the perception of digital difficulty is strongly age-dependent, with a clear generational divide concentrated at the upper age strata. Younger patients (especially those aged 25–30) approach digital technologies as a familiar component of everyday life and therefore report low difficulty, whereas patients over 60 years experience a much steeper learning curve when interacting with digital interfaces in the dental care pathway [43,44]. This generational pattern is consistent with the findings reported among clinicians [19] and managers [20], but the magnitude of the gap is substantially more pronounced among patients, particularly at the upper age range. Unlike clinicians and managers, who function within professional environments that incentivize continuous digital adaptation, patients aged over 60 may lack the routine institutional exposure that gradually consolidates digital competence. This difference suggests that AI integration in dental practice cannot rely on the assumption of digital literacy among older patient cohorts and must instead be designed with explicit accessibility safeguards, intuitive interfaces, and human-mediated alternatives for those who may otherwise be excluded from the benefits of digital transformation.

#### 3.3.2. Analysis of the Perception of the Degree of Difficulty in Relation to Education

**Figure 4:**
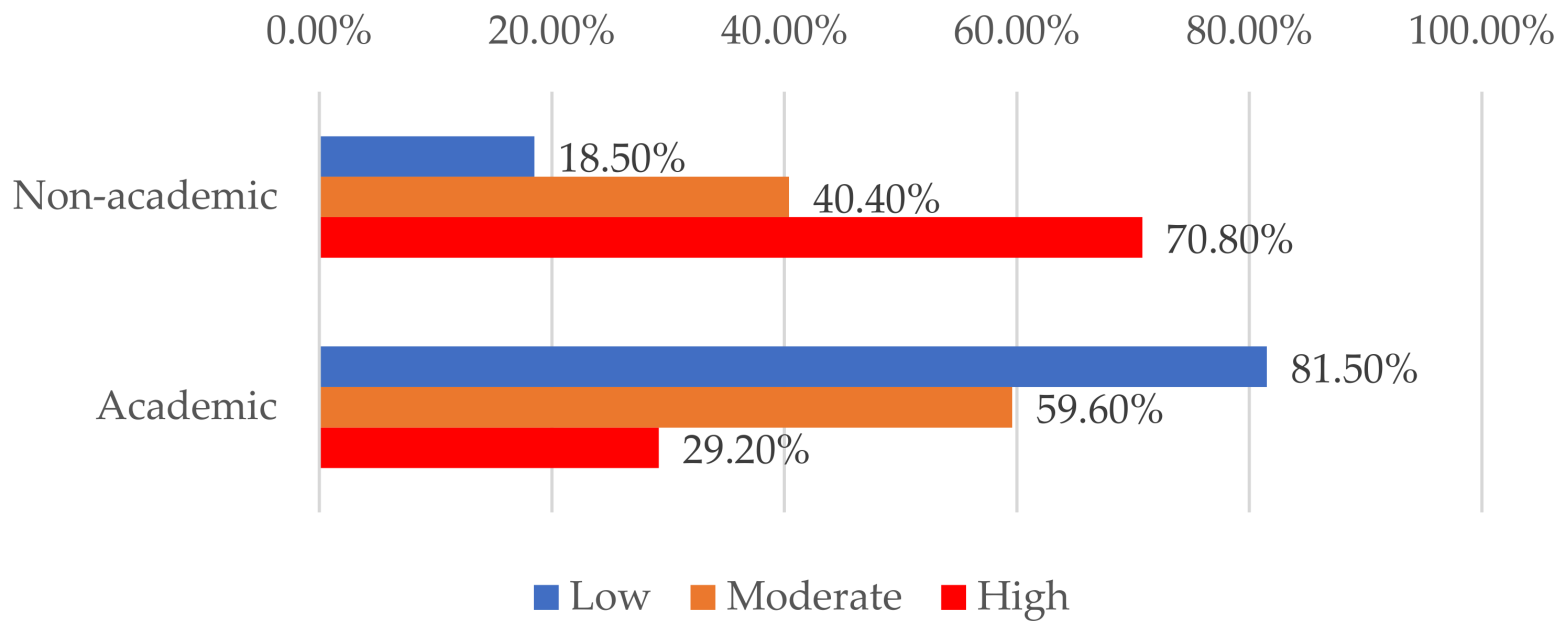
Distribution of patients according to educational level and degree of difficulty.

The data in Table 3 and Figure 4 represent the distribution of patients in terms of educational level (academic vs. non-academic) and degree of difficulty. The differences observed between groups according to Fisher’s Exact Test were significant (*p* < 0.001), and Bonferroni-corrected comparisons of column proportions showed that patients with non-academic education were disproportionately represented among those reporting a high level of difficulty (70.8% of the high-difficulty subgroup, compared with 40.4% of the moderate-difficulty and 18.5% of the low-difficulty subgroups), whereas patients with academic education were disproportionately represented among those reporting a low or moderate level of difficulty (81.5% and 59.6%, respectively, versus 29.2% of the high-difficulty subgroup). Read within educational groups, high difficulty was reported by 27.4% of non-academic patients compared with 5.1% of academic patients, while low difficulty was reported by 35.5% and 70.3%, respectively.

This finding represents one of the most distinctive contributions of the present study and introduces a dimension that was not explicitly analyzed in our previous investigations on clinicians [19] and managers [20], whose professional cohorts were homogeneously composed of academically educated individuals. Among dental patients, educational background emerges as a robust predictor of perceived digital difficulty, with non-academic patients facing significantly steeper challenges than their academically educated counterparts. This pattern likely reflects multiple underlying mechanisms: differential exposure to digital tools in professional and personal environments, varying levels of health literacy, and distinct capacities to critically navigate complex informational ecosystems. Importantly, the educational divide observed here may also act as a proxy for socio-economic and cultural factors that shape access to and engagement with digital health technologies. From a practical perspective, these results suggest that AI integration in dental practice must be accompanied by educational and communicational support tailored to patients with different educational profiles, in order to prevent the consolidation of a digital inequality that would otherwise undermine the principles of equitable access to care.

### 3.4. The Perceived Degree of Availability

Beyond the perceived level of difficulty, patients’ availability to engage with new AI-based and digital solutions represents a complementary dimension that captures the proactive readiness to integrate these technologies into their dental care experience. Among the 200 respondents, 93 (46.5%) reported a high level of availability, 75 (37.5%) reported a moderate level, and 32 (16%) reported a low level. This distribution suggests that a substantial majority of patients are open to AI-supported solutions in dental practice, although a meaningful subset remains reserved. A detailed analysis according to age and educational background yielded the following results.

#### 3.4.1. Analysis of Availability in Relation to Age

**Figure 5:**
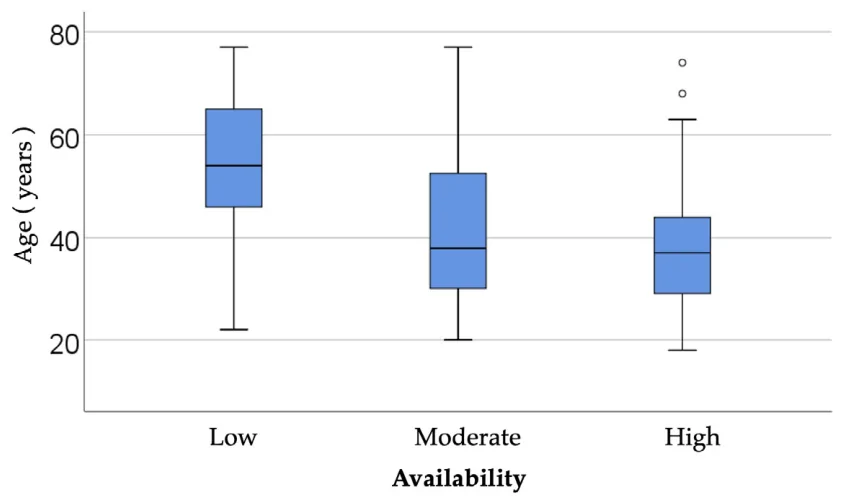
Comparison of patients’ age in relation to reported availability.

The data in Table 4 and Figure 5 represent the comparison of patients’ age in relation to their reported availability. The differences observed between groups according to the Kruskal–Wallis H test were significant (*p* < 0.001), and Dunn–Bonferroni post-hoc tests show that patients who reported low availability were significantly older (median = 54, IQR = 45.5–65) compared to patients who reported moderate availability (median = 38, IQR = 30–53) (*p* = 0.001) or high availability (median = 37, IQR = 29–44) (*p* < 0.001); the differences between moderate- and high-availability groups were not significant (*p* = 0.235), (Kruskal–Wallis H(2) = 26.852, *p* < 0.001, ε^2^ = 0.135, a moderate effect).

The categorical age-group analysis (Table 5) confirms this pattern. According to Fisher’s Exact Test, the differences across age categories were significant (*p* < 0.001). Bonferroni-corrected comparisons of column proportions indicated that patients aged 51–55 years were over-represented among those reporting moderate availability relative to high availability (13.3% of the moderate-availability subgroup vs. 2.2% of the high-availability subgroup), whereas patients aged over 60 years were over-represented among those reporting low availability (37.5% of the low-availability subgroup vs. 8% and 3.2% of the moderate- and high-availability subgroups). Within the over-60 group itself, low availability was the predominant response (57.1%).

This age-related pattern is consistent with the diffusion of the innovations literature, which describes a stronger predisposition among younger groups to explore and adopt new solutions, while older groups adopt a more cautious attitude filtered through established routines. The similarity with the patterns reported among clinicians [19] and managers [20] is notable, but with one important nuance: in the patient cohort, the upper-age cutoff effect is considerably more dramatic, with low availability reported by 57.1% of patients aged over 60 (a group that alone accounted for 37.5% of all respondents in the low-availability category), a much sharper drop than what was observed in the professional cohorts of our previous studies. This generational asymmetry underscores the need for AI implementation strategies that explicitly account for the heterogeneity of patient receptiveness across age strata and that provide reassuring, gradually introduced digital touchpoints for older patients who may otherwise feel excluded from technologically advanced dental services.

#### 3.4.2. Analysis of Availability in Relation to Education

**Figure 6:**
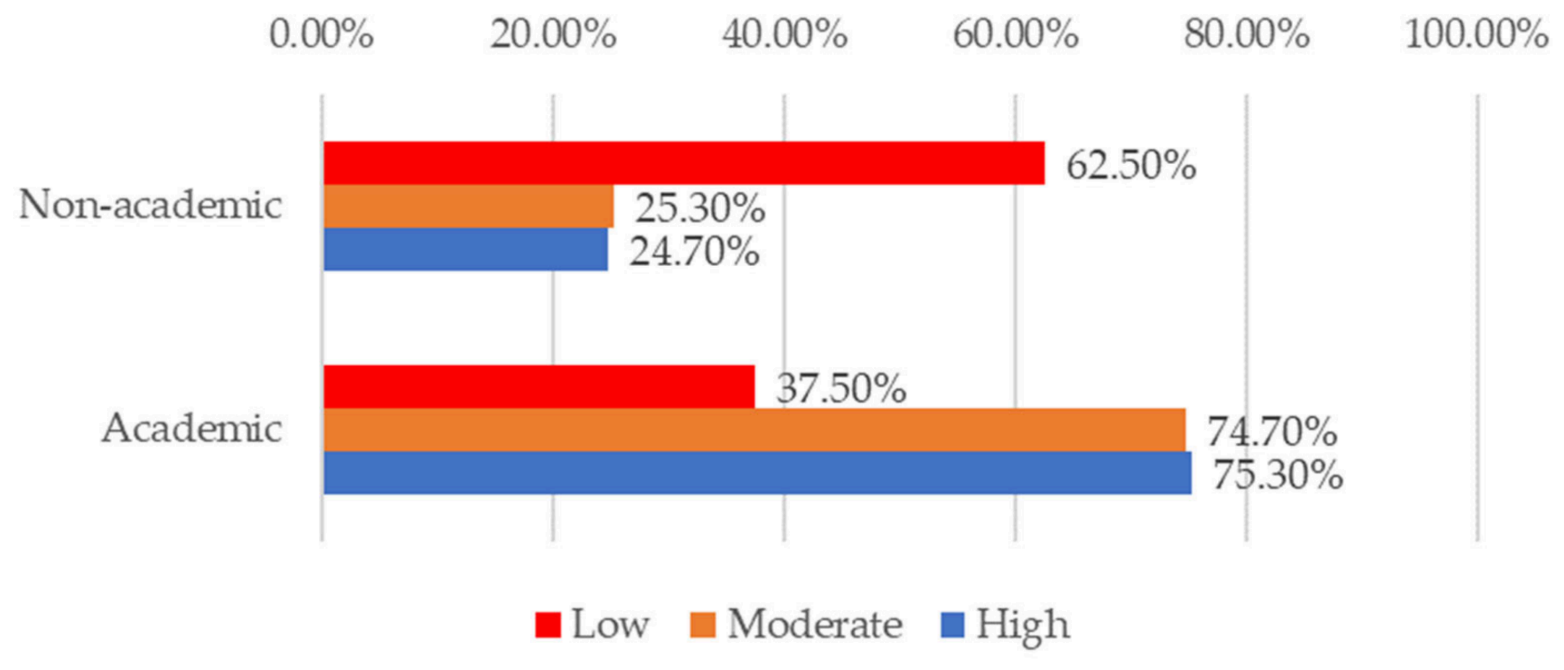
Distribution of patients according to educational level and availability.

The data in Table 6 and Figure 6 represent the distribution of patients in terms of educational level and availability. The differences observed between groups according to Fisher’s Exact Test were significant (*p* < 0.001), and Bonferroni-corrected comparisons of column proportions showed that non-academic patients were disproportionately represented among those with low availability (62.5% of the low-availability subgroup, compared with 25.3% and 24.7% of the moderate- and high-availability subgroups), whereas academic patients were disproportionately represented among those with moderate or high availability (74.7% and 75.3%, respectively, versus 37.5% of the low-availability subgroup). Read within educational groups, low availability was reported by 32.3% of non-academic patients compared with 8.7% of academic patients, whereas high availability was reported by 37.1% and 50.7%, respectively.

The strong educational gradient observed for availability mirrors the pattern previously identified for perceived difficulty and represents a robust, original finding of the present study. Patients with academic education demonstrate substantially higher openness to integrating AI-supported solutions into their dental care, while patients with non-academic education are markedly more reserved. This difference likely reflects the combined influence of greater exposure to digital tools in professional settings, higher health literacy, and a more developed capacity to critically evaluate the benefits and limits of new technologies. This educational divide reinforces, for the dimension of availability, the same practical concern already noted for perceived difficulty: without targeted educational and communicational support, less academically educated patients risk being inadvertently excluded from the benefits of digital transformation.

### 3.5. The Use of Digital Tools in Current Practice

The use of digital tools in the patient’s current interactions with dental services represents the operational counterpart of the perceptual dimensions analyzed above and provides a concrete measure of how far digitalization has already entered routine patient experience. Among the 200 respondents, 137 (68.5%) reported currently using digital tools in connection with their dental care (such as online booking systems, appointment-reminder applications, electronic communication with the dental practice, and digital access to medical information), while 63 (31.5%) reported no current use. A detailed analysis according to age and educational background yielded the following results.

#### 3.5.1. Analysis of the Use of Digital Tools in Relation to Age

**Figure 7:**
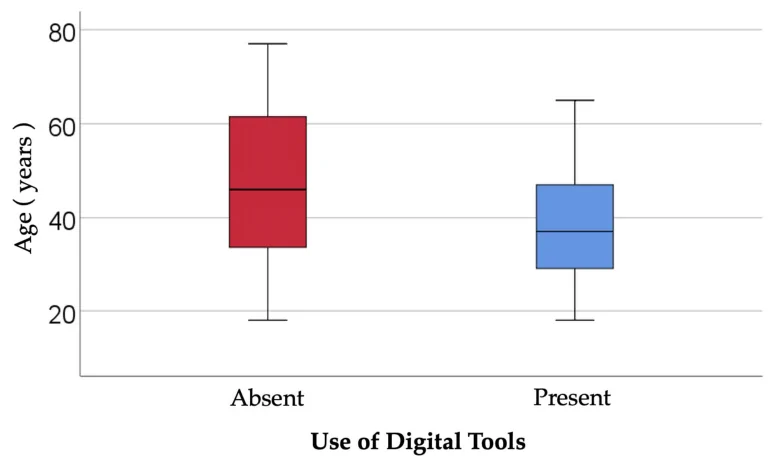
Comparison of patients’ age in relation to the use of digital tools.

The data in Table 7 and Figure 7 represent the comparison of patients’ age in relation to the use of digital tools. The differences observed between groups according to the Mann–Whitney U test were significant (*p* = 0.001), such that patients who reported currently using digital tools were significantly younger (median = 37, IQR = 29–47) compared to patients who do not use digital tools (median = 46, IQR = 33–62), (Mann–Whitney U = 3030, Z = −3.382, *p* = 0.001, r = 0.239).

The categorical age-group analysis (Table 8) provides a more granular picture. According to Fisher’s Exact Test, the differences across age categories were significant (*p* < 0.001). Bonferroni-corrected comparisons of column proportions indicated that patients aged over 60 years were markedly over-represented among non-users of digital technology (27% of the non-user subgroup vs. 2.9% of the user subgroup). Within the over-60 group itself, 81% reported no current use of digital tools.

This pattern aligns with the generational findings reported for clinicians [19] and managers [20] and confirms that age remains a primary determinant of digital engagement across all three perspectives of the dental healthcare triad. From the patient’s perspective, however, here too the over-60 effect is more pronounced than in the professional cohorts, with 27% of these patients reporting no current use of digital tools. This generational concentration suggests that the older patient segment represents the demographic group most at risk of being functionally excluded from the benefits of digital and AI-supported dental care, unless specific accessibility measures are deliberately implemented.

#### 3.5.2. Analysis of the Use of Digital Tools in Relation to Education

**Figure 8:**
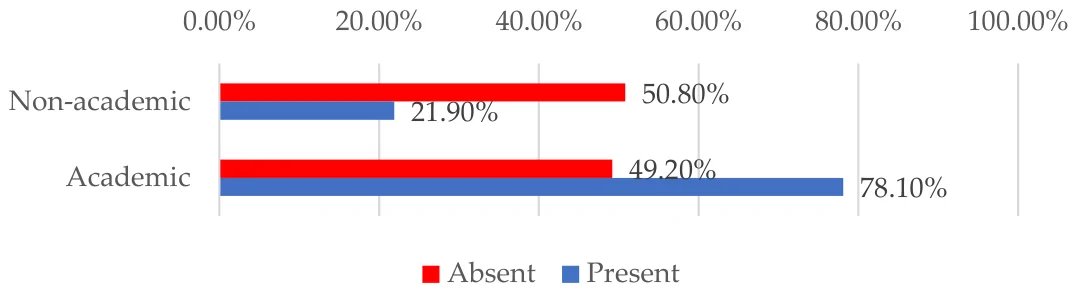
Distribution of patients according to educational level and use of digital tools.

The data in Table 9 and Figure 8 represent the distribution of patients in terms of educational level and use of digital tools. The differences observed between groups according to Fisher’s Exact Test were significant (*p* < 0.001). Current use of digital tools was considerably more prevalent within the academic group than within the non-academic group (77.5% vs. 48.4%); equivalently, academic patients were significantly over-represented among current users of digital tools, constituting 78.1% of all users. Non-academic patients, by contrast, were more evenly divided between users and non-users.

This finding completes the consistent pattern of educational differentiation observed across all three dimensions analyzed in this study (perceived difficulty, availability, and current use) and confirms that educational background is a robust predictor of digital engagement among dental patients. The 29.1 percentage-point difference in the current use of digital tools between the academic (77.5%) and non-academic (48.4%) groups, calculated within each educational group, represents one of the most striking gradients identified in the present research and reinforces the practical implications already outlined: this confirms, now for the operational dimension of current use, the educational divide observed across all three measures, underscoring the need for the educationally tailored implementation strategies outlined above.

### 3.6. Description of Interest Scores in Relation to Digitalization

Beyond the analysis of operational dimensions (perceived difficulty, availability, and current use of digital tools) and the five subdomains of the dental care pathway, two integrative interest scores were constructed to capture the attitudinal architecture through which patients evaluate the broader implications of AI integration and digitalization in dental practice. These two scores were derived from exploratory Principal Component Analysis (PCA) applied to all items in the survey, followed by assessment of internal consistency through Cronbach’s alpha, without any claim of formal construct validation. The two emerging components represent complementary but distinct dimensions of patient attitudes that, taken together, outline the full attitudinal profile of the dental patient confronting digital transformation.

The first integrative score is the overall digital prudence score (Cronbach’s α = 0.893, λ = 7.888), reflecting an excellent level of internal consistency. This score captures the following traits: major concern for data security, skepticism toward automation when it is perceived as displacing logistics or human contact, and awareness of current systemic shortcomings in the digital ecosystem. A higher value indicates a clearly prudent attitudinal profile, in which the patient is genuinely interested in digitalization only when data security is guaranteed and when digitalization effectively solves their time- and care-management problems, while simultaneously fearing data theft or a cooling of the personal relationship with the clinician. The median observed value was 3.24 (IQR = 2.82–3.61), indicating a moderate-to-elevated level of digital prudence.

The second integrative score is the overall cost-sensitivity and technological-sustainability score (Cronbach’s α = 0.651, λ = 2.457), reflecting only a moderate (marginal) level of internal consistency; this value is lower than that of the digital prudence score and, while acceptable for exploratory research, indicates that the construct is measured with limited internal consistency, so that results involving this score should be interpreted with corresponding caution (see Limitations, Section 3.10). This score captures the following traits: fear of increased dental fees, perception of the opportunity cost associated with rapid technological obsolescence (which is expected to force clinics into recurring investments and price increases), and limited accessibility due to financial barriers (the absence of modern diagnostics being closely linked to affordability). A higher value reflects a pragmatic, cost-conscious patient profile who recognizes the value of technology but whose principal concern is that digitalization may render dental care prohibitively expensive, with implementation and maintenance costs being transferred to the patient. The median observed value was 3.00 (IQR = 2.44–3.44), indicating a moderate level of cost sensitivity and concern for technological sustainability. The internal logic of this score illustrates the reorientation procedure described in Section 2. Its positively loaded items express, in explicit terms, the anticipation that the costs of digital technology will be transferred to the patient, as in the item concerning the high costs of purchasing, updating and maintaining technology and the consequent increase in fees (loading = 0.720), the item on the high initial investment and maintenance costs of new technologies resulting in increased tariffs (0.607), and the corresponding item in the follow-up subdomain (0.584). The negatively loading items express the same underlying pragmatic orientation in the opposite direction: patients concerned primarily with the cost of digitalization tend not to regard the reduction in personal interaction with the clinician (−0.592) or a possible malfunction of automated recall systems (−0.512) as salient challenges, since they consider the existing arrangements adequate and further investment in these directions unnecessary; similarly, interest in transforming complex medical information into AI-generated visual formats (−0.359) runs counter to a cost-sensitive orientation, as greater technological sophistication implies greater expense. These items were therefore reverse-scored on the basis of their content, so that higher values of the composite score consistently denote a more pragmatic, cost-conscious profile. An equivalent conceptual examination was carried out for the digital prudence score.

The results in Table 10 outline the profile of a dental patient whose attitude toward digitalization and AI integration is ***dual in nature***: genuinely open to the benefits of technological transformation, but simultaneously cautious about its informational, relational, and financial implications. The moderate-to-elevated median value of the digital prudence score suggests that most patients do not reject digitalization itself, but instead approach it through a layered filter of concerns regarding data security, the preservation of the human dimension of care, and the operational reliability of digital systems. They are willing to engage with digital and AI-supported solutions, but only under conditions of perceived informational and relational safety. The moderate median value of the technological-sustainability score complements this profile by adding a clear pragmatic dimension: even when patients accept digitalization in principle, their support is conditioned by the expectation that the costs of implementation will not be transferred to them through increased fees and that digital tools will remain durable and affordable over time.

This dual attitudinal profile (*open but prudent, pragmatic but cost-conscious*) represents a clear contrast to the profiles previously identified among clinicians [19] and managers [20]. Clinicians approached AI primarily through the lens of clinical efficiency and diagnostic support, while expressing reservations about data security and the doctor–patient relationship. Managers approached AI through an efficiency-driven and risk-aware framework, favoring incremental integration. Patients, by contrast, introduce a third distinct logic: they evaluate AI through the combined lens of ***informational safety*** (digital prudence) and ***economic sustainability*** (cost sensitivity), with both dimensions converging on the protection of the trust-based relationship with the dental practice.

### 3.7. Correlations Between Interest Scores and Demographic Characteristics

#### 3.7.1. Correlation Between Digital Prudence Score and Age

**Figure 9:**
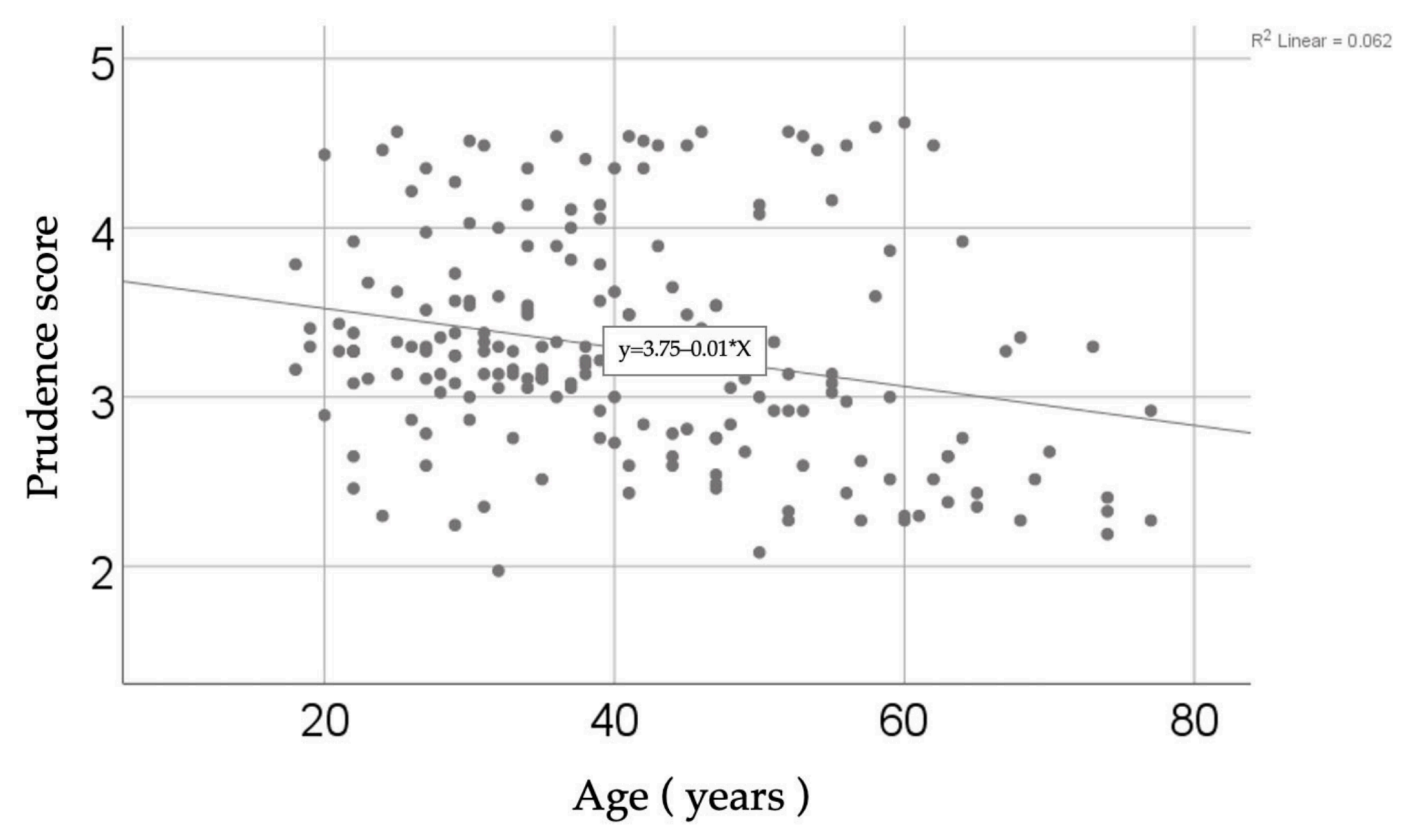
Correlation between digital prudence score and age (* represents a multiplication sign).

The data in Table 11 and Figure 9 represent the correlation between the digital prudence score and patient age. The correlation observed was significant and negative, of low magnitude (Spearman’s rho = −0.260, *p* < 0.001), indicating that younger patients were significantly more frequently associated with higher digital prudence scores, and vice versa.

This finding is one of the most theoretically interesting results of the present study and at first sight may appear counterintuitive. One might intuitively expect older patients, who report higher perceived difficulty, lower availability, and less frequent use of digital tools, to also display higher digital prudence. Instead, the data reveal the opposite pattern: ***younger patients, who are more digitally engaged, are also the most digitally prudent***. This pattern suggests that digital prudence is not driven primarily by unfamiliarity or fear of technology, but rather by a more informed and critical awareness of the actual risks associated with AI and digital systems. Younger patients, who are exposed to digital media on a daily basis, are likely to have a more nuanced understanding of data-security risks, the limitations of algorithmic recommendations, and the potential for digital interfaces to displace meaningful human interaction. Older patients, by contrast, may be less aware of these specific risks precisely because they engage less frequently with digital systems, and their reservations about AI integration may be expressed instead through perceived difficulty rather than through articulated prudence. This interpretation will be developed further in the Discussion Section, particularly in connection with the proposed concept of informational VUCA.

#### 3.7.2. Correlation Between Technological Sustainability Score and Age

**Figure 10:**
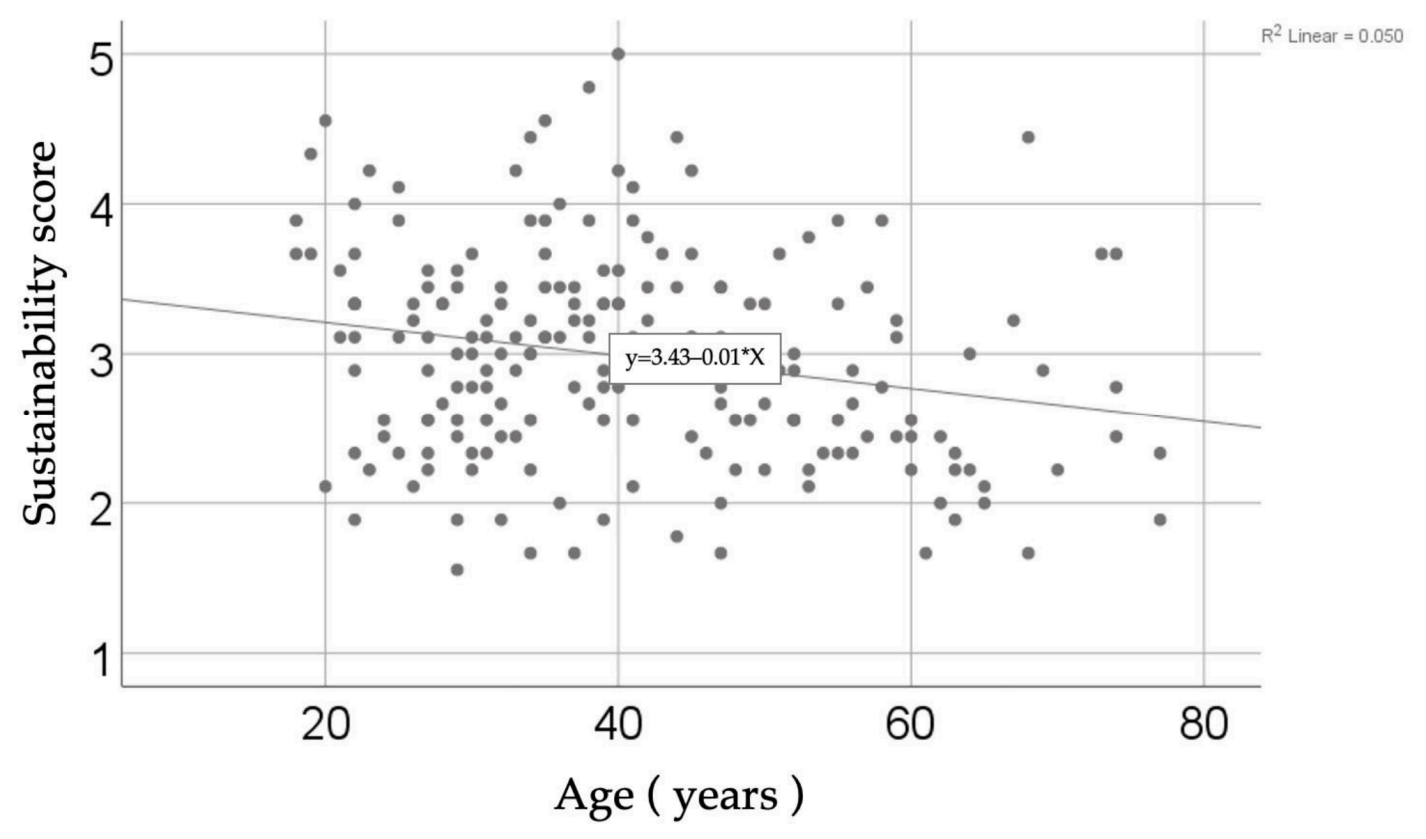
Correlation between technological sustainability score and age (* represents a multiplication sign).

The data in Table 12 and Figure 10 represent the correlation between the technological sustainability score and patient age. The correlation observed was significant and negative, of low magnitude (Spearman’s rho = −0.208, *p* = 0.003), indicating that younger patients were significantly more frequently associated with higher technological sustainability scores, and vice versa.

The pattern observed for technological sustainability mirrors that of digital prudence: younger patients also score higher on concerns related to cost and the financial implications of digital transformation. This convergence is consistent with the interpretation outlined above and suggests that digitally engaged patients are not only more aware of informational risks but also more attuned to the economic dynamics of technological adoption. They appear to recognize that AI integration in dental practice carries implementation and maintenance costs that may eventually be reflected in service fees, and they express this awareness through a more articulated cost-sensitivity profile. Older patients, who engage less directly with digital systems, may not have developed a comparable awareness of these financial dynamics, and their concerns about dental care affordability may be expressed through other channels not captured by this specific score.

#### 3.7.3. Comparison of Digital Prudence Score by Educational Level

The data in Table 13 represent the comparison of the digital prudence score in relation to educational level. The differences observed between groups according to the Mann–Whitney U test were significant (*p* < 0.001), with academically educated patients scoring significantly higher on digital prudence (median = 3.30, IQR = 3.05–3.90) compared to non-academically educated patients (median = 2.95, IQR = 2.59–3.28), (Mann–Whitney U = 2574, Z = −4.50, *p* < 0.001, r = 0.318).

The educational gradient observed for digital prudence further confirms the interpretive framework developed in the previous subsections: digital prudence increases with informational exposure and critical capacity. Academically educated patients, who have higher health literacy and more developed analytical skills, are better equipped to recognize and articulate the specific risks associated with AI and digital systems. Non-academic patients, while expressing higher operational difficulty and lower availability, do not necessarily translate these concerns into a structured prudence profile, but rather into more diffuse reservations about digital engagement. This pattern reinforces the importance of educational interventions tailored to different patient profiles, in order to ensure that all categories of patients can develop an informed and balanced attitude toward AI integration in dental care.

#### 3.7.4. The Dual Attitudinal Profile: Digital Prudence and Technological Sustainability

The dual attitudinal profile identified through the two integrative interest scores, digital prudence and technological sustainability, outlines a patient figure who is open to AI integration but conditionally engaged. Patients support digitalization when it visibly addresses concerns about data security, preserves the human dimension of care, and remains financially sustainable. They are wary, however, of solutions that increase costs without corresponding improvements in care quality, that displace meaningful human interaction, or that introduce informational complexity they are not equipped to navigate. This conditional engagement represents a distinct logic that differs from the efficiency-driven framework of managers [20] and the clinical-decision-support framework of clinicians [19], and it calls for AI implementation strategies that explicitly address informational, relational, and economic dimensions in an integrated manner.

The counterintuitive finding that digital prudence is positively correlated with digital engagement, meaning that the most digitally engaged patients are also the most prudent, is particularly important from a practical standpoint. It suggests that ***patient education in AI matters cannot be approached as a simple effort to overcome resistance***, since the most informed patients are precisely those who express the most articulated concerns. Rather, the practical task is to engage with these concerns as legitimate, evidence-informed contributions to the design of AI integration strategies, and to develop communication practices that transparently address the risks of data security, algorithmic limitations, and relational dilution that informed patients clearly perceive. This perspective will be developed further in the subsequent sections through the lens of Maslow’s hierarchy of needs and the proposed framework of informational VUCA.

### 3.8. Maslow’s Hierarchy of Needs Applied to the Dental Patient in the Age of AI

The attitudinal profile that emerged from the present study, combining openness with informed prudence and a clear hierarchy of priorities across the care pathway, cannot be fully understood through operational variables alone; it calls for a framework that captures the full architecture of patient motivation. Abraham Maslow’s classical hierarchy of needs [45], proposed in 1943 and later refined [46,47], remains one of the most enduring such frameworks. Building on Maslow [45] and on the complementary motivational theory of McClelland [48,49], we propose an interpretive mapping of the dental patient’s needs across five hierarchical levels, indicating for each how AI integration may strengthen or threaten it and which subdomain of the care pathway it may correspond to. We present this mapping as a theoretical interpretation intended to organize and give structure to the pattern of results observed in our study, rather than as a model empirically tested or validated by the survey data. The correspondences we draw between hierarchical levels of need and care-pathway subdomains are interpretive proposals offered to make sense of the observed distribution of patient interest and concern, and should be read as hypothesis-generating rather than as confirmatory findings.

#### 3.8.1. The Foundational Level: Physiological Safety, Data Integrity, and the Diagnostic Encounter

At the foundational level lie the most basic concerns of physical safety during the medical act and the integrity of personal data, whose defining need is the safe and successful outcome of the therapeutic act [21,22]. This level is operationalized through the diagnostic subdomain, which drew the highest patient interest (median = 3.30, IQR = 2.70–3.90). Diagnosis is the most consequential phase of the encounter, the moment at which the clinician’s competence is implicitly judged and the foundation of trust for the whole therapeutic process is established [50]. Patients value AI-supported diagnostic tools as credibility-enhancing instruments that reinforce, rather than replace, clinical judgment [35]. Yet the same level has a dual face: the high digital prudence score (median = 3.24, IQR = 2.82–3.61), most pronounced among the digitally engaged, signals clear awareness of new risks of data-security vulnerability and algorithmic opacity [51,52]. Acceptance is therefore conditional on credible guarantees of data security and clinical accuracy, with AI introduced transparently as a decision-support resource rather than an autonomous diagnostic agent.

#### 3.8.2. The Safety and Security Level: Therapeutic Predictability, Financial Sustainability, and Treatment Planning

The second level concerns safety, security, and predictability, which in dental care split into the therapeutic safety of the proposed plan and the financial security that care remains affordable over time. It corresponds to the treatment planning subdomain (median = 2.86, IQR = 2.14–3.29), notable for moderate interest but the highest dispersion across responses: patients value decision-support tools that clarify alternatives, yet fear that algorithmic recommendations may dilute the personalized, judgment-based character of the plan. The financial dimension is captured by the technological sustainability score (median = 3.00, IQR = 2.44–3.44), reflecting concern that implementation and maintenance costs, together with rapid obsolescence, may be passed on through higher fees and may exclude vulnerable patients [53,54]. This level is therefore intertwined with both clinical and economic concerns, aligning closely with the risk-aware framework of managers [20].

#### 3.8.3. The Social and Affiliation Level: Relational Continuity and Follow-Up Care

The third level encompasses social and affiliation needs, the patient’s wish for a continuous, personalized relationship with the clinician and the practice, closely related to McClelland’s need for affiliation [48,49]. It corresponds to the follow-up (dispensarization) subdomain (median = 3.00, IQR = 2.64–3.55), the temporal extension of the doctor–patient relationship beyond the immediate encounter. AI suits this level well, since personalized recall systems, adaptive reminders, and accessible channels can reinforce continued connection without depending on real-time interaction. The same level, however, carries one of our sample’s most consistent concerns: that excessive automation may dilute the human dimension of care [51]. AI here succeeds only when designed as an extension of, not a substitute for, the human relationship, preserving direct clinician–patient contact at the critical junctures of care.

#### 3.8.4. The Esteem and Recognition Level: Feedback as Validation

The fourth level concerns esteem, recognition, and validation, the patient’s wish to be acknowledged as an active participant and to be heard about their experience, related to McClelland’s need for achievement and recognition [48,49]. It corresponds to the feedback subdomain (median = 3.11, IQR = 2.78–3.67), one of the most distinctive findings of this study. Unlike clinicians [19] and managers [20], who ranked feedback as low priority, patients treat the post-treatment phase as a critical moment in which the relational symmetry of the encounter is temporarily restored: the recipient of care becomes the source of information that shapes future care. AI-supported feedback can serve this need through structured, personalized channels, but only when designed with attention to its relational meaning rather than as impersonal data collection. The practical implication is clear: in patient-centered AI implementation, feedback should be treated as a primary, not a secondary, subdomain.

#### 3.8.5. The Self-Actualization Level: Informed Autonomy and the Gateway of Care

At the apex lies self-actualization, the patient’s capacity for informed autonomy: to weigh therapeutic alternatives and participate as a knowledgeable partner [21,22], related to McClelland’s need for power and control [48,49] in its constructive form as agency over one’s own health. Paradoxically, the scheduling subdomain, which drew the lowest interest (median = 2.70, IQR = 2.00–3.30), also belongs here, as the gateway through which the patient enters the care system. Its low score reflects not indifference but an expectation that automation remain unobtrusive and preserve human mediation for sensitive cases. Notably, the patients who most actively pursue this level, those with higher education and greater digital engagement, are also those who most clearly articulate its boundaries. This introduces a key nuance into the classical framework: acceptance of AI at this level depends not on trust alone but on an equilibrium between trust and critical awareness [49,50], so that informed patients become genuine partners only when their critical perspective is incorporated into implementation.

#### 3.8.6. Synthesis: The Integrated Hierarchy of Patient Needs in AI-Mediated Dental Care

**Figure 11:**
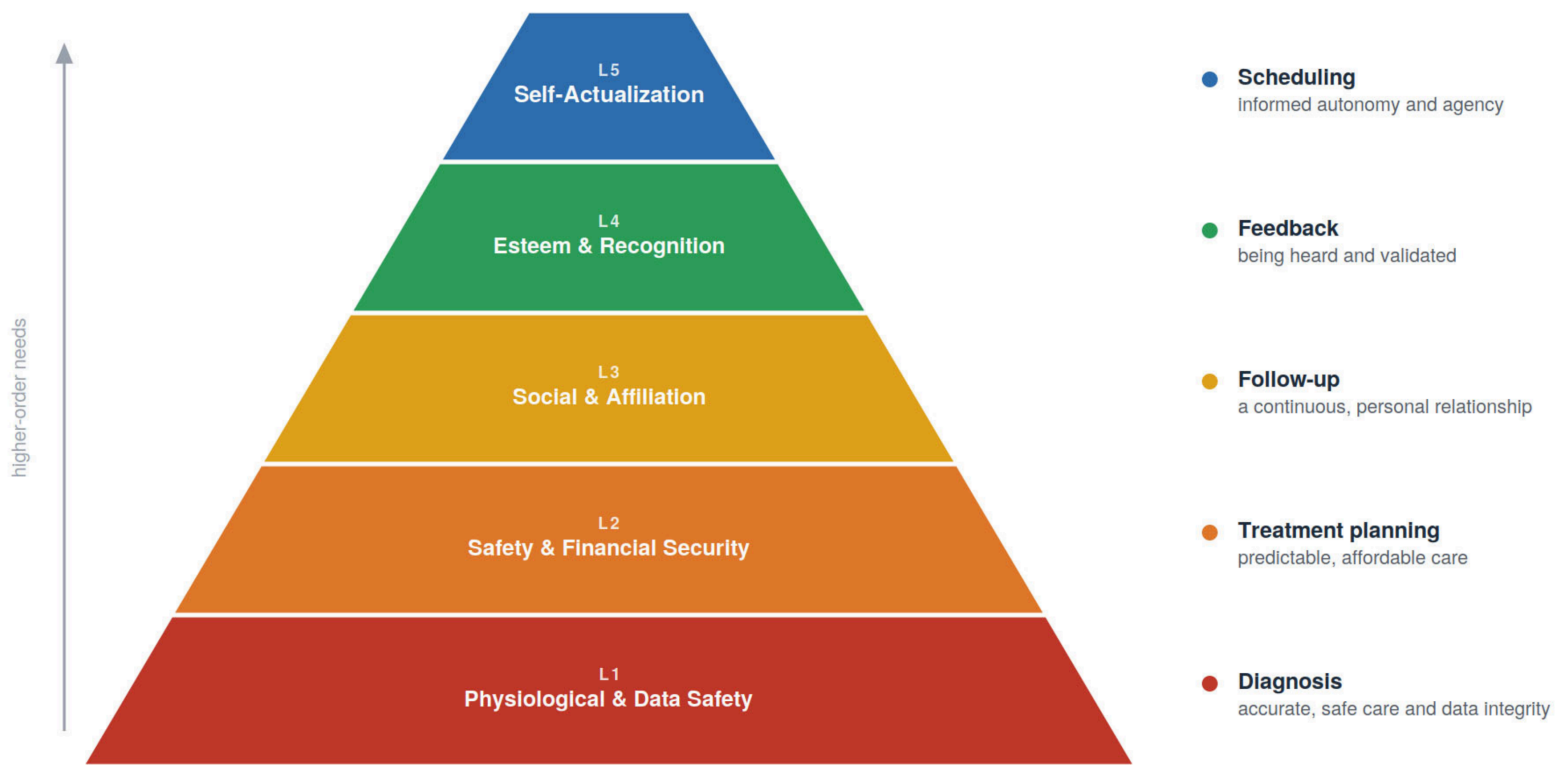
Maslow’s hierarchy of needs applied to the dental patient in the context of AI integration, with associated opportunities and risks at each level.

Taken together, the five levels may be read as an integrated framework for how AI integration intersects with the full architecture of patient motivation (Figure 11). Each level maps onto a specific subdomain: diagnosis at the foundational level, treatment planning at the safety level, follow-up at the affiliation level, feedback at the esteem level, and scheduling at the self-actualization level, which may suggest an interpretive structure for the patient’s hierarchy of needs in the age of AI-mediated dental care. This correspondence is offered as a heuristic organizing device consistent with the present data rather than as an empirically validated hierarchy.

This integrated reading of the patient’s motivational hierarchy may provide a conceptual bridge between the empirical results of the present study and the framework developed next, the proposed concept of **informational VUCA**, which addresses the specific informational challenges that patients face when navigating AI-mediated content.

### 3.9. Informational VUCA: A Proposed Conceptual Framework for Patient-Centered AI Implementation

**Figure 12:**
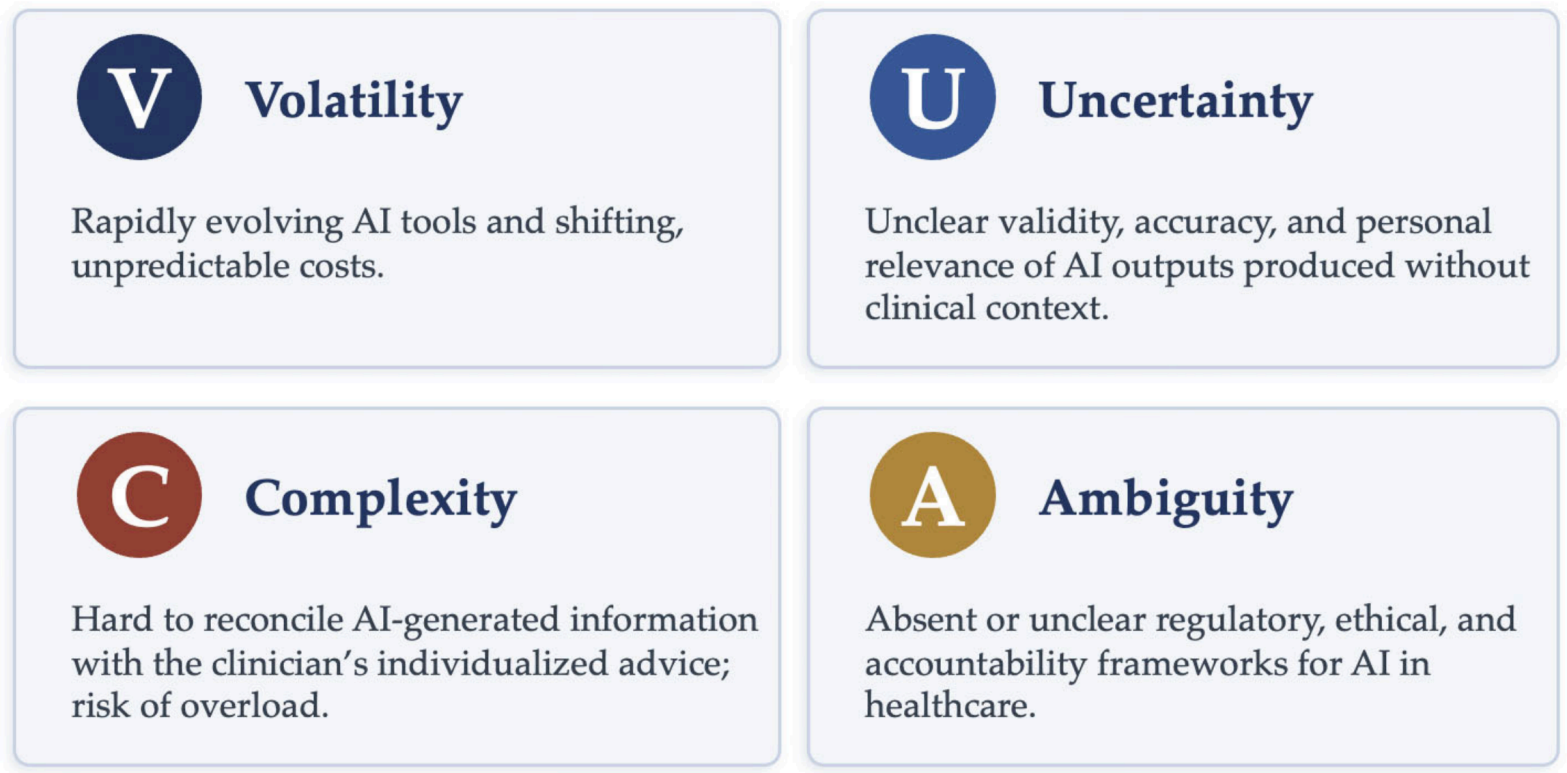
The Informational VUCA framework applied to patient-centered AI implementation in dental care, showing its four dimensions: volatility, uncertainty, complexity, and ambiguity.

Building on the present findings and on the Maslow-based interpretation above, we propose a conceptual model we designate as Informational VUCA, a structured framework for the informational challenges patients face when AI and digital technologies mediate the dental care pathway. It adapts the established VUCA paradigm from strategic management [55,56], already extended to healthcare and the doctor–patient relationship under uncertainty [17], to the patient-facing informational space, where the line between clinically validated content and AI-generated, algorithmically mediated information is increasingly hard to draw. Its empirical anchor is one of our most distinctive findings, the positive association between digital engagement and digital prudence (Section 3.7): the most digitally engaged patients also score highest on prudence. This counterintuitive pattern, replicated across age, education, and the three operational dimensions of engagement, may indicate not digital anxiety but rather informational exposure and critical awareness, and can be interpreted as suggesting that the more patients engage with AI, the more clearly they perceive its risks, uncertainties, and ambiguities [51,52]. Given the cross-sectional design of the study, this reading remains associative and does not permit causal inference. The framework structures this awareness through four dimensions (Figure 12). We propose this framework as an interpretative, hypothesis-generating lens for organizing the patient-facing informational challenges observed in our data, rather than as a claim that extends beyond what the present exploratory findings can directly support; each of its four dimensions is therefore linked, where possible, to specific empirical results of the study. It is important to underline that the four dimensions of this frame-work—volatility, uncertainty, complexity, and ambiguity—were not measured as formal, operationalized constructs in the present study; rather, each is a conceptual descriptor that we link, where possible, to specific empirical indicators already reported, and the framework should be read as an interpretive scaffold for these observations, not as a set of validated measurement dimensions.

#### 3.9.1. Informational Volatility

**Volatility** denotes the rapid, hard-to-predict pace of change in an environment [53,54]. In the patient-facing space it captures the rapid evolution of AI tools, algorithmic recommendations, and digital health platforms, which can render previously valid information obsolete within short timeframes. Beyond this informational dimension, volatility also has a less frequently discussed economic face. Volatility has a second, rarely discussed face, the economic volatility of AI integration. The continuous emergence of new tools generates recurring acquisition, licensing, training, and maintenance costs for clinics that are ultimately transferred to patients through higher fees [53,54], so that the pace of innovation may paradoxically restrict rather than expand access to care. This may be reflected empirically in the technological sustainability score (median = 3.00, IQR = 2.44–3.44) and by the negative correlations of age with the prudence and sustainability scores (Spearman’s ρ = −0.260, *p* < 0.001, and ρ = −0.208, *p* = 0.003, respectively), which may suggest that younger patients, immersed in rapid technological turnover, perceive both the informational and the financial instability most clearly.

#### 3.9.2. Informational Uncertainty

**Uncertainty** refers to situations where available information cannot support accurate forecasting [55,56]. Here it captures the patient’s difficulty in judging the validity, accuracy, and personal applicability of AI-generated outputs, which are produced without case-specific context; lacking medical training, the patient is structurally disadvantaged in assessing relevance. A possible empirical indication of this dimension is the high dispersion of responses in the treatment planning subdomain (IQR = 2.14–3.29), the stage where the gap between generalized recommendations and personalized judgment is most exposed. Uncertainty thus may underscore the irreducible role of the clinician in translating generalized outputs into clinically meaningful decisions for the individual patient.

#### 3.9.3. Informational Complexity

**Complexity** characterizes environments of intricate, interconnected processes [55,56]. Here it captures the cognitive burden of integrating AI-generated information with the clinician’s individualized advice, with other digital sources, and with the patient’s own prior knowledge. This dimension may be reflected in the strong educational gradient in the digital prudence score (academic median = 3.30 vs. non-academic = 2.95, *p* < 0.001) [57,58], which can be interpreted as suggesting that academically educated patients navigate this complexity more readily, whereas those with non-academic education struggle to integrate multiple streams. This may indicate a population-level vulnerability, namely that informational complexity could disproportionately affect subgroups with lower informational literacy, with consequences for equitable access to AI-supported care.

#### 3.9.4. Informational Ambiguity

**Ambiguity** refers to situations of multiple or contradictory meanings [55,56]. Here it captures the absence of clear regulatory, ethical, and accountability frameworks for AI in healthcare [59,60], which leaves patients uncertain about the legitimacy and oversight of the information they consume: who is responsible if a recommendation is wrong, what governs the algorithm’s validation, and how their data is stored and protected. The elevated prudence seen precisely among educated, digitally engaged patients may therefore be interpreted not as technophobia but as a calibrated response to recognized ambiguity, consistent with calls for comprehensive regulatory frameworks that clarify accountability, transparency, and patient protection [61,62,63].

#### 3.9.5. The Integrative Logic of Informational VUCA

Together, the four dimensions outline the informational landscape patients navigate in AI-mediated dental care and may offer a coherent explanation for the layered, conditional attitudinal profile our data reveal. AI implementation should therefore aim not only at efficiency and accuracy but at actively reducing informational VUCA at the patient interface: clear communication on the role and limits of AI tools (ambiguity), structured patient-education materials that contextualize outputs (complexity), explicit clinician validation of AI-generated information (uncertainty), and stable, well-maintained platforms (volatility). The framework also points to a possible principal vulnerability: patients with lower education and older patients may face a compounded form of informational VUCA that could exclude them from the benefits of digital transformation [57,58]. Taken together, these considerations point to a single overarching principle for practice: AI implementation in dentistry should be patient-centered, transparent, and explicitly adapted to patients’ differing levels of age, education, and digital literacy, so that the benefits of digital transformation extend to all patient groups rather than widening existing inequalities.

These tensions are not unique to dentistry. Analogous challenges of explainability, regulatory harmonization, data governance, and equitable access appear across AI-intensive medical domains, from AI-assisted liquid-biopsy interpretation in colorectal oncology [64] to machine learning prediction of outcomes such as hospital length of stay after surgery [65], suggesting that informational VUCA, though derived from the dental patient’s perspective, may capture a more general feature of AI-mediated care across the medical specialties. Both conceptual contributions advanced in this section, the Maslow-based hierarchy of patient needs and the Informational VUCA framework, should therefore be regarded as exploratory interpretive constructs generated by, rather than confirmed through, the present survey data. They are intended to organize the observed patterns into testable propositions and to guide subsequent research, and their validity will need to be examined in larger, multicentre, and preferably longitudinal designs employing dedicated measurement instruments.

### 3.10. Limitations

This study has several limitations that should be acknowledged. First, and most importantly for the interpretation of our findings, the study employed an exploratory cross-sectional design based on a purposive/convenience sample of patients recruited in Bucharest and the surrounding areas. Because recruitment relied on a pragmatic, in-person distribution strategy rather than a closed sampling frame, a formal response rate could not be calculated, and the sample cannot be regarded as statistically representative of the broader Romanian dental patient population; because participation depended on in-person recruitment from patients already in contact with dental services, some degree of selection bias toward more engaged patients cannot be excluded. Together with the geographic concentration of respondents, these features limit the generalizability of the findings to other regions of Romania and to international contexts, where socio-economic, cultural, and healthcare-system factors may shape patient attitudes in different ways. Accordingly, the present results should be interpreted as exploratory and hypothesis-generating rather than fully representative, and as characterizing the specific population surveyed rather than as broadly representative of dental patients in Romania or elsewhere. Second, although the sample size of 200 patients is consistent with the exploratory design of the study and with the corresponding cohorts of our previous clinician [19] and managerial [20] investigations, it remains limited for the purpose of supporting fine-grained subgroup analyses. Third, the rapidly evolving nature of AI and digital technologies in healthcare means that patient attitudes captured at a specific point in time may shift as new tools, regulatory frameworks, and public-health communications reshape the informational landscape.

Fourth, although the measurement instrument was designed as part of a coordinated, cross-perspective research project and showed good internal consistency for its principal construct, it underwent only preliminary, exploratory validation; formal content-validity assessment, expert-panel review, independent pilot testing, and cross-cultural psycho-metric validation were not undertaken and remain objectives for future work. Fifth, the technological sustainability score showed only moderate internal consistency (Cronbach’s α = 0.651), lower than that of the digital prudence score (α = 0.893); while acceptable for an exploratory study, this indicates that the construct is measured less robustly, and the corresponding findings should be regarded as preliminary. Sixth, most of the reported analyses are bivariate; because age, education, digital exposure, and area of residence are themselves interrelated, these associations may be mutually confounded, and the present data—given the sample size and the small counts in several outcome categories—do not support stable adjusted models, so adequately powered multivariable modelling is identified as a priority for future confirmatory work. Seventh, the sample was drawn predominantly from urban, academically educated patients (84.5% urban and 69% with academic education) recruited in person from among active dental patients, which introduces a potential for selection bias and further constrains generalizability to rural populations, to patients with lower educational attainment, and to individuals with less frequent contact with dental services. Finally, the percentages reported in the contingency tables are column proportions, and the associated post-hoc comparisons therefore describe the relative over- or un-der-representation of a given subgroup within a response category rather than the distribution of responses within that subgroup; within-group proportions are provided in the text wherever the distinction is material to the interpretation.

## 4. Conclusions

The integration of artificial intelligence into dental practice represents a structural transformation of the patient experience that extends well beyond the introduction of new technological tools. The central message of the present study is that dental patients are broadly open to AI integration, but conditionally so: they welcome it specifically when it enhances diagnostic confidence, preserves the clinician–patient relationship, ensures data security, and remains economically sustainable, and they become cautious when these conditions are not credibly met. From the patient’s perspective, AI is therefore not a self-evident solution to be uncritically adopted, but a complex informational reality to be navigated with informed care. Because these findings derive from an exploratory, convenience-based sample recruited in a single metropolitan region, they should be understood as characterizing the surveyed population and as generating hypotheses for larger, multi-regional studies rather than as broadly representative of dental patients in general.

The patient’s attitudinal profile is shaped by a layered hierarchy of needs that spans the full architecture of human motivation, from foundational concerns about physical and data safety, through the security and predictability of care, the affiliation-based continuity of the doctor–patient relationship, the recognition expressed through structured feedback, to the highest-order need for informed autonomy in therapeutic decision-making. At each level of this hierarchy, AI offers genuine opportunities and introduces specific risks, and patient acceptance is conditional on implementation strategies that strengthen the opportunities while credibly mitigating the risks.

The present study contributes two conceptual frameworks that may support patient-centered AI implementation in dental practice, both of which should be understood as exploratory interpretive proposals rather than as validated models. First, the Maslow-based hierarchy of patient needs in AI-mediated dental care may provide a structured map for understanding how digital tools intersect with the full architecture of patient motivations. Second, the Informational VUCA framework, proposed in this study as an original conceptual contribution, identifies the specific informational challenges that patients may face when navigating AI-mediated content and provides actionable guidance for reducing informational volatility, uncertainty, complexity, and ambiguity at the patient interface. Both frameworks are advanced as hypothesis-generating instruments that require empirical validation in future research.

**Figure 13:**
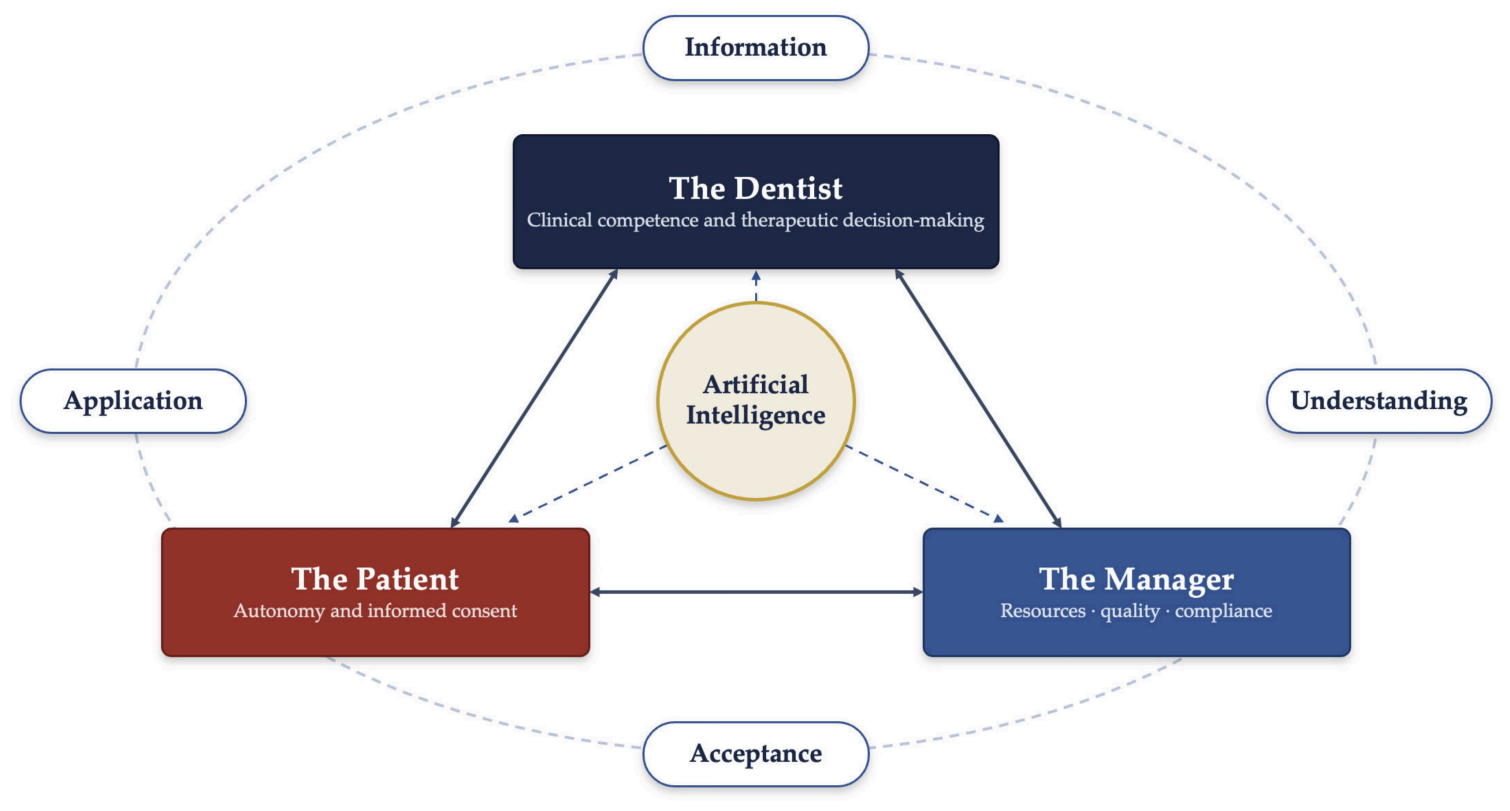
AI integration in dental practice.

Taken together with our previous studies on dental clinicians [19] and dental clinic managers [20], the present research completes a triangulated picture of AI integration in dental practice and confirms the central role of the patient as the integrative connector of the dental healthcare triad (Figure 13). The implementation of AI in dental practice should therefore be approached not as a purely technical or administrative project, but as an integrated socio-technical transformation that requires the coordinated engagement of clinicians, managers, and patients, and that explicitly addresses the informational, relational, ethical, and economic dimensions of digital change. Only under these conditions can AI fulfill its genuine promise: to support, rather than to replace, the human dimensions of dental care, and to strengthen, not erode, the trust-based relationship that remains the foundation of meaningful therapeutic practice.

In summary, this cross-sectional study characterizes the dental patient as a conditionally engaged actor in the process of digital transformation: broadly receptive to AI-supported care, yet consistently prudent in proportion to the degree of digital engagement, educational attainment, and informational exposure. The principal scientific contributions of the study are threefold: an empirically derived hierarchy of patient interest across the five subdomains of the dental care pathway, in which diagnosis and feedback emerge as priority domains; the identification of a dual attitudinal profile combining openness with digital prudence and cost sensitivity, together with the counterintuitive association between digital engagement and prudence; and the formulation of two exploratory conceptual frameworks, the Maslow-based hierarchy of patient needs and Informational VUCA, intended to translate these findings into testable propositions for subsequent research. Taken together, these results indicate that the patient-centered implementation of AI in dental practice depends less on the technical performance of the tools themselves than on the transparency, relational continuity, and informational support with which they are introduced into clinical practice.

## Figures and Tables

**Table 1:** Comparison of patients’ age according to perceived degree of difficulty.

Difficulty	Mean ± SD	Median (IQR)	Mean Rank	*p* *
Low (N = 119)	35.88 ± 10.56	34 (28–43)	80.50	<0.001
Moderate (N = 57)	44.30 ± 13.66	42 (35–54.5)	116.46	
High (N = 24)	58.13 ± 13.68	63 (50.5–68.75)	161.73	

* Kruskal–Wallis H Test.: H(2) = 45.431, *p* < 0.001, ε^2^ = 0.228 (large effect).

**Table 2:** Distribution of patients according to age category and degree of difficulty.

Age/Difficulty	Low (*n*, %)	Moderate (*n*, %)	High (*n*, %)	*p* *
18–24 years	15 (12.6%)	6 (10.5%)	0 (0%)	<0.001
25–30 years	29 (24.4%)	2 (3.5%)	1 (4.2%)	
31–35 years	21 (17.6%)	7 (12.3%)	2 (8.3%)	
36–40 years	17 (14.3%)	10 (17.5%)	0 (0%)	
41–45 years	16 (13.4%)	5 (8.8%)	1 (4.2%)	
46–50 years	9 (7.6%)	8 (14%)	2 (8.3%)	
51–55 years	6 (5%)	6 (10.5%)	3 (12.5%)	
56–60 years	4 (3.4%)	7 (12.3%)	2 (8.3%)	
>60 years	2 (1.7%)	6 (10.5%)	13 (54.2%)	

* Fisher’s Exact Test. Cramér’s V = 0.46. Percentages represent column proportions (i.e., the proportion of each response category accounted for by each group); within-group distributions are reported in the text.

**Table 3:** Distribution of patients according to educational level and degree of difficulty.

Education/Difficulty	Low (*n*, %)	Moderate (*n*, %)	High (*n*, %)	*p* *
Non-academic	22 (18.5%)	23 (40.4%)	17 (70.8%)	<0.001
Academic	97 (81.5%)	34 (59.6%)	7 (29.2%)	

* Fisher’s Exact Test. Cramér’s V = 0.38. Percentages represent column proportions (i.e., the proportion of each response category accounted for by each group); within-group distributions are reported in the text.

**Table 4:** Comparison of patients’ age according to availability.

Availability	Mean ± SD	Median (IQR)	Mean Rank	*p* *
Low (N = 32)	53.28 ± 15.10	54 (45.5–65)	146.19	<0.001
Moderate (N = 75)	40.88 ± 13.38	38 (30–53)	100.55	
High (N = 93)	36.76 ± 11.37	37 (29–44)	84.74	

* Kruskal–Wallis H Test.: H(2) = 26.852, *p* < 0.001, ε^2^ = 0.135 (moderate effect).

**Table 5:** Distribution of patients according to age category and availability.

Age/Availability	Low (*n*, %)	Moderate (*n*, %)	High (*n*, %)	*p* *
18–24 years	1 (3.1%)	5 (6.7%)	15 (16.1%)	<0.001
25–30 years	3 (9.4%)	15 (20%)	14 (15.1%)	
31–35 years	1 (3.1%)	14 (18.7%)	15 (16.1%)	
36–40 years	2 (6.3%)	8 (10.7%)	17 (18.3%)	
41–45 years	1 (3.1%)	6 (8%)	15 (16.1%)	
46–50 years	5 (15.6%)	5 (6.7%)	9 (9.7%)	
51–55 years	3 (9.4%)	10 (13.3%)	2 (2.2%)	
56–60 years	4 (12.5%)	6 (8%)	3 (3.2%)	
>60 years	12 (37.5%)	6 (8%)	3 (3.2%)	

* Fisher’s Exact Test. Cramér’s V = 0.383. Percentages represent column proportions (i.e., the proportion of each response category accounted for by each group); within-group distributions are reported in the text.

**Table 6:** Distribution of patients according to educational level and availability.

Education/Availability	Low (*n*, %)	Moderate (*n*, %)	High (*n*, %)	*p* *
Non-academic	20 (62.5%)	19 (25.3%)	23 (24.7%)	<0.001
Academic	12 (37.5%)	56 (74.7%)	70 (75.3%)	

* Fisher’s Exact Test. Cramér’s V = 0.297. Percentages represent column proportions (i.e., the proportion of each response category accounted for by each group); within-group distributions are reported in the text.

**Table 7:** Comparison of patients’ age according to usage of digital tools.

Use of Digital Tools	Mean ± SD	Median (IQR)	Mean Rank	*p* *
Absent (N = 63)	46.90 ± 16.52	46 (33–62)	120.90	<0.001
Present (N = 137)	38.21 ± 11.66	37 (29–47)	91.12	

* Mann–Whitney U Test.: U = 3030, Z = −3.382, *p* = 0.001, r = 0.239.

**Table 8:** Distribution of patients according to age category and use of digital instruments.

Age/Use	Absent (*n*, %)	Present (*n*, %)	*p* *
18–24 years	4 (6.3%)	17 (12.4%)	<0.001
25–30 years	8 (12.7%)	24 (17.5%)	
31–35 years	7 (11.1%)	23 (16.8%)	
36–40 years	8 (12.7%)	19 (13.9%)	
41–45 years	4 (6.3%)	18 (13.1%)	
46–50 years	7 (11.1%)	12 (8.8%)	
51–55 years	4 (6.3%)	11 (8%)	
56–60 years	4 (6.3%)	9 (6.6%)	
>60 years	17 (27%)	4 (2.9%)	

* Fisher’s Exact Test. Cramér’s V = 0.382. Percentages represent column proportions (i.e., the proportion of each response category accounted for by each group); within-group distributions are reported in the text.

**Table 9:** Distribution of patients according to educational level and use of digital instruments.

Education/Use	Absent (*n*, %)	Present (*n*, %)	*p* *
Non-academic	32 (50.8%)	30 (21.9%)	<0.001
Academic	31 (49.2%)	107 (78.1%)	

* Fisher’s Exact Test. Cramér’s V = 0.29. Percentages represent column proportions (i.e., the proportion of each response category accounted for by each group); within-group distributions are reported in the text.

**Table 10:** Description of Interest Scores in Relation to Digitalization.

Score	Mean ± SD	Median (IQR)
Digital Prudence Score	3.281 ± 0.646	3.243 (2.817–3.614)
Technological Sustainability Score	2.978 ± 0.691	3.000 (2.444–3.444)

**Table 11:** Correlation between digital prudence score and age.

Correlation	*p* *
Digital Prudence Score × Age	rho = −0.260, *p* < 0.001

* Spearman’s rho correlation coefficient.

**Table 12:** Correlation between technological sustainability score and age.

Correlation	*p* *
Technological Sustainability Score × Age	rho = −0.208, *p* = 0.003

* Spearman’s rho correlation coefficient.

**Table 13:** Comparison of digital prudence score in relation to educational level.

Education	Mean ± SD	Median (IQR)	Mean Rank	*p* *
Non-academic	2.98 ± 0.50	2.95 (2.59–3.28)	73.02	<0.001
Academic	3.42 ± 0.66	3.30 (3.05–3.90)	112.85	

* Mann–Whitney U Test.: U = 2574, Z = −4.502, *p* < 0.001, r = 0.318.

## Data Availability

The original contributions presented in this study are included in the article. Further inquiries can be directed to the corresponding author.
